# Genomic characterization of IDH-mutant astrocytoma progression to grade 4 in the treatment setting

**DOI:** 10.1186/s40478-023-01669-9

**Published:** 2023-11-06

**Authors:** Kirsi J. Rautajoki, Serafiina Jaatinen, Anja Hartewig, Aliisa M. Tiihonen, Matti Annala, Iida Salonen, Masi Valkonen, Vili Simola, Elisa M. Vuorinen, Anni Kivinen, Minna J. Rauhala, Riikka Nurminen, Kendra K. Maass, Sirpa-Liisa Lahtela, Arja Jukkola, Olli Yli-Harja, Pauli Helén, Kristian W. Pajtler, Pekka Ruusuvuori, Joonas Haapasalo, Wei Zhang, Hannu Haapasalo, Matti Nykter

**Affiliations:** 1 Prostate Cancer Research Center, Faculty of Medicine and Health Technology, Tampere University and Tays Cancer Centre, Tampere University Hospital, Tampere, Finland; 2 Tampere Institute for Advanced Study, Tampere University, Tampere, Finland; 3 Institute of Biomedicine, University of Turku, Turku, Finland; 4 Department of Neurosurgery, Tampere University Hospital and Tampere University, Tampere, Finland; 5 Faculty of Medicine and Health Technology, Tampere University and Tays Cancer Centre, Tampere, Finland; 6 Hopp Children’s Cancer Center Heidelberg (KiTZ), Heidelberg, Germany; 7 Division of Pediatric Neuro Oncology, German Cancer Research Center, German Cancer Consortium (DKTK), Heidelberg, Germany; 8 Department of Pediatric Oncology, Hematology, Immunology and Pulmonology, Heidelberg University Hospital, Heidelberg, Germany; 9 Department of Oncology, Tampere University Hospital and Tays Cancer Centre, Tampere, Finland; 10 Institute for Systems Biology, Seattle, WA USA; 11 Fimlab Laboratories Ltd., Tampere University Hospital, Tampere, Finland; 12 Cancer Genomics and Precision Oncology, Wake Forest Baptist Comprehensive Cancer Center, Winston-Salem, NC USA

**Keywords:** Diffuse glioma, Secondary glioblastoma, Non-homologous end-joining, Microhomology-mediated end-joining, Homologous recombination repair, Longitudinal analysis, Cancer genomics, RNA-sequencing

## Abstract

As the progression of low-grade diffuse astrocytomas into grade 4 tumors significantly impacts patient prognosis, a better understanding of this process is of paramount importance for improved patient care. In this project, we analyzed matched IDH-mutant astrocytomas before and after progression to grade 4 from six patients (discovery cohort) with genome-wide sequencing, 21 additional patients with targeted sequencing, and 33 patients from Glioma Longitudinal AnalySiS cohort for validation. The Cancer Genome Atlas data from 595 diffuse gliomas provided supportive information. All patients in our discovery cohort received radiation, all but one underwent chemotherapy, and no patient received temozolomide (TMZ) before progression to grade 4 disease. One case in the discovery cohort exhibited a hypermutation signature associated with the inactivation of the *MSH2* and *DNMT3A* genes. In other patients, the number of chromosomal rearrangements and deletions increased in grade 4 tumors. The cell cycle checkpoint gene *CDKN2A,* or less frequently *RB1*, was most commonly inactivated after receiving both chemo- and radiotherapy when compared to other treatment groups. Concomitant activating *PDGFRA/MET* alterations were detected in tumors that acquired a homozygous *CDKN2A* deletion. *NRG3* gene was significantly downregulated and recurrently altered in progressed tumors. Its decreased expression was associated with poorer overall survival in both univariate and multivariate analysis. We also detected progression-related alterations in *RAD51B* and other DNA repair pathway genes associated with the promotion of error-prone DNA repair, potentially facilitating tumor progression. In our retrospective analysis of patient treatment and survival timelines (n = 75), the combination of postoperative radiation and chemotherapy (mainly TMZ) outperformed radiation, especially in the grade 3 tumor cohort, in which it was typically given after primary surgery. Our results provide further insight into the contribution of treatment and genetic alterations in cell cycle, growth factor signaling, and DNA repair-related genes to tumor evolution and progression.

**Supplementary Information:**

The online version contains supplementary material available at 10.1186/s40478-023-01669-9.

## Introduction

Diffuse gliomas are a broad class of brain tumors originating from brain glial cells. They are diagnosed in approximately 100,000 patients each year worldwide [1] and are associated with substantial mortality despite comprising less than 2% of all newly diagnosed cancers [2]. Adult diffuse gliomas are subdivided into three molecular subtypes that differ in their prognosis and age of onset: primary isocitrate dehydrogenase wild-type (IDHwt) glioblastoma (GB), IDH-mutant (IDHmut) astrocytoma, and IDHmut oligodendroglioma. Both of IDHmut gliomas carry a hotspot missense mutation in *IDH1* or *IDH2* [3].

Many grade 2–3 IDHmut astrocytomas eventually progress to grade 4, previously referred to as secondary glioblastomas [3]. Because tumor progression drastically worsens patient prognosis, understanding the mechanisms leading to tumor progression is important for the development and implementation of optimal treatment strategies. In addition to surgery, grade 2–3 diffuse gliomas are typically subjected to DNA-damaging treatments, such as radiation, chemotherapy, or both, which may also contribute to tumor evolution [4]. They provide selective pressure on tumor cells and induce changes in DNA, leading to new genetic alterations. For example, temozolomide (TMZ), typically used against grade 4 tumors, is a DNA-damaging agent associated with a characteristic hypermutator phenotype and radiotherapy has been linked to the increase of genomic deletions [5–8]. Radiation is the only known environmental risk factor for developing low-grade gliomas [9], and it increases the risk of homozygous *CDKN2A* deletion [8], which is frequently observed in recurrent grade 4 IDHmut astrocytomas and is associated with increased proliferation [10].

In this study, we investigated the progression of diffuse IDHmut astrocytomas from grade 2–3 to grade 4 using whole-genome sequencing (WGS) and strand-specific RNA-sequencing (RNA-seq). All patients in our discovery cohort received radiation, and most underwent chemotherapy; however, none had received TMZ before progression. The number of chromosomal rearrangements and DNA copy number alterations (CNA) increased notably in all progressed cases except in one hypermutator case that carried a biallelic loss of the *MSH2* gene. Accumulated structural variants (SVs) were accompanied by homozygous loss of *CDKN2A* or *RB1* and activating chromosomal alterations in growth factor receptor genes. Additional patient cohorts were used for targeted analysis and validation of results.

## Methods

### In-house study cohorts

An experienced neuropathologist evaluated the formalin-fixed, paraffin-embedded (FFPE) tumor samples and determined the histopathological type and grade according to the criteria presented by the World Health Organization (WHO) [3]. For the discovery cohort, matched frozen tumor samples obtained before and after progression to grade 4 astrocytoma were collected from six patients (Additional file 1: Table S1). Matched blood control DNA was not available. The primary tumors in the discovery cohort were diagnosed between 1984 and 2008. The analyzed tumors were operated 1993–2010. The tumor content in the samples was estimated from frozen tumor sections stained with hematoxylin and eosin (H and E), after which every third section was used for RNA isolation and the others for DNA isolation. DNA was isolated from frozen samples using the QIAamp DNA Mini kit (QIAGEN) with RNase treatment. RNA was isolated with the mirVana™ miRNA Isolation Kit (Life Technologies), dividing small (< 200 nucleotides) and large RNA (> 200 nucleotides) molecules into separate fractions. For targeted sequencing analysis in the validation cohort, matched brain tumor samples were collected from 21 patients whenever possible. Tumors included in the targeted sequencing analysis were operated between 1984 and 2012. In two cases, only grade 4 tumors were analyzed. The grading of the first relapse of TG15 was changed from grade 3 to grade 4 due to *CDKN2A* deletion observed in the targeted sequencing. We did not need to change the grading of other samples in our in-house cohorts based on *CDKN2A* status. Most of the samples were from FFPE sections, and DNA was isolated using either the truXTRAC FFPE DNA Kit (Covaris) or the GeneRead DNA FFPE Kit (Qiagen).

### Whole-genome and targeted DNA sequencing

For WGS, library construction and WGS were performed using a TruSeq DNA kit (Illumina) at the Beijing Genomics Institute (BGI), Hong Kong. Samples were sequenced with the Illumina HiSeq 2000 technology using paired-end (PE90) sequencing, with at least 90 Gb of clean data per sample. The pursued sequencing depth was 30 × . For targeted sequencing, libraries were prepared with TruSeq® Custom Amplicon Kit v1.5 (Illumina) and sequenced with Illumina MiSeq technology using PE150 sequencing. Data were aligned against the University of California, Santa Cruz (UCSC) genome browser hg38 human genome assembly (hg19 for targeted sequencing) [11] using Bowtie 2 (version 2.2.9). Duplicate reads were masked using Samblaster (version 0.1.22).

### DNA methylation analysis

Genome-wide DNA methylation profiling was performed using an Illumina Infinium HumanMethylation EPIC Kit. DNA methylation-based molecular classification was performed as previously described [12]. For comparison, the DNA methylation profiles of the presented cases were visualized as a t-distributed stochastic neighbor embedding (tSNE) plot, together with an in-house Heidelberg Brain Tumor Methylation Classifier cohort of 64,891 samples from different tumor entities. The Heidelberg Brain Tumor Methylation Classifier is based on 2,682 central nervous system (CNS) tumors, representing 82 distinct tumor methylation classes (https://www.molecularneuropathology.org) [12]. tSNE clustering was conducted on the M-values of the 10,000 most variable CpGs using the Rtsne and Rspectra packages.

### RNA-sequencing analysis

Library construction and sequencing of the RNA samples were performed at BGI using the Illumina HiSeq 2000 technology. Transcriptome sequencing was performed for the large RNAs using a strand-specific 140–160 bp short insert library protocol, and at least 8 Gb of clean paired-end (PE90) data were obtained per library. Sequencing reads were aligned using STAR version 2.7.0 against the human genome assembly GRCh38 using GENCODE version 30 annotations. Gene expression was determined using the featureCounts algorithm in the Rsubread package version 1.34.6. Read count normalization and paired differential expression (DE) between genes before and after progression were computed using paired DESeq2. Genes with an adjusted p-value < 0.05 based on Wald’s test and a log2 fold change (log2FC) > 1 were considered significant. ConsensusPathDB [13] was utilized for Gene Ontology (GO) term enrichment analysis by using the enrichment analysis (*p* < 0.01) and fold changes of differentially expressed genes. GO levels 3–5 were considered, excluding the “cellular component” ontology. Terms with a q-value < 0.05 were considered as significant. Additionally, a list of genes sorted according to adjusted p-value was uploaded to GOrilla [14], and significant GO terms for “Process” as well as “Function” were calculated (q < 0.05, based on minimum hypergeometric statistics). The obtained list of GO terms was filtered to contain only terms reported by both tools, as well as terms with at least ten genes measured in the dataset. The GO terms were summarized and plotted using Revigo [15] and R package treemap (version 2.4–3).

### Gene set activities

Gene sets of interest, including hypoxia, proliferation, homologous recombination repair (HRR), non-homologous end-joining (NHEJ), microhomology-mediated end-joining (MMEJ), and Fanconi Anemia (FA), were collected from the MSigDB database [16] or literature [17, 18]. Initial hypoxia and proliferation gene sets were further filtered to contain only the genes whose expression correlates with each other in The Cancer Genome Atlas (TCGA) diffuse glioma cohort (Pearson and Spearman’s correlation ≥ 0.4 between all genes). This resulted in four hypoxia signature genes (*VEGFA, ADM, PDK1*, and *CA9*) out of the ten previously reported hypoxia-related genes (Winter et al*.* [17]) as well as 125 and 122 cell cycle S- and G2/M-phase genes, respectively (Additional file 1: Table S2). Activity z-scores [19] were calculated for each gene set across the discovery and TCGA diffuse glioma cohorts. Heatmaps and violin plots for activity scores as well as other data were created with R packages ggplot2 (versions 3.3.5 and 3.4.0), gplots (version 3.1.3), and ComplexHeatmap (version 2.6.2).

### Mutation analysis

Somatic mutations were required to have at least three unique supporting reads and a variant allele fraction (VAF) of 10% or higher in the WGS data. In the targeted amplicon sequencing data with high depth, we required 30 supporting reads and VAF ≥ 10%. To filter out technical artifacts, only variants where the VAF significantly differed between cohort samples were included in the analysis. To evaluate this, we used a chi-square goodness of fit test to calculate the p-value for the probability that the observed distribution of alternate and reference allele read counts occurred by chance under the null hypothesis of samples having identical underlying VAFs for the mutation. Finally, mutations reported in the gnomAD v3.0 database of human germline variants were filtered out. Specific mutations were separately confirmed from targeted or RNA-seq data for case TG06, for which we did not have WGS data, and also in case we were unable to make a confident call due to low sequencing coverage in WGS. The *IDH1* mutation status of the TG14 grade 3 tumor was verified using IHC staining due to low coverage of the location in targeted sequencing data. For mutation signature analysis, the tri-nucleic context of the mutations was evaluated [20] and the numbers of each substitution type were plotted using an R package MutationalPatterns (version 3.0.1).

### Copy number analysis

In WGS analysis, the number of aligned sequencing reads was counted for each 1000 bp window tile of the genome. To exclude sequencing technology and sequence alignment biases, coverage log ratios were calculated relative to a normal kidney tissue sample WGS data as previously described [21]. Finally, the coverage log ratios were median-decimated 200-fold to suppress noise and exported as IGV (Integrative Genomics Viewer) tracks for visual interpretation. In targeted sequencing analysis, read depths were normalized to regions lacking copy number alterations in IDHmut astrocytomas and DNA copy numbers were determined from violin plots visualizing coverage log ratios of analyzed genes for each patient. Only homozygous deletions and amplifications were called.

### Chromosomal rearrangement analysis

Chromosomal rearrangements in the discovery cohort were detected from the WGS data using Breakfast (commit e94e922). Rearrangements were required to be supported by at least five unique reads and any rearrangements identified in over 30% of the samples were filtered out. Clonal rearrangements were acquired by filtering the fraction of reads supporting the rearrangements to the total number of reads, which was required to be at least 0.2 either at the junction-spanning reads or inside the span of the rearrangement read pairs on both sides of the breakpoint. In addition, rearrangements with a breakpoint within the 200 bp range in the matched grade 2–3 or grade 4 samples were considered clonal in both samples. Circos v.0.69–9 was used to visualize chromosomal rearrangements across the chromosomes, and ggplot2 (version 3.3.5) to visualize single chromosomes. Microhomology in rearrangements was determined as previously described [8]; by comparing the 5′ end of the breakpoint to the 3’ rearranged sequence up to 20 bp with 2 bp as a minimum length for microhomology. Intrachromosomal rearrangements < 2 Mb were determined as focal.

### Recurrently rearranged genes

Public topologically associating domain (TAD) boundaries from neural progenitor cells [22] were used to define the genes whose expressions were possibly altered due to rearrangements unique to grade 4 tumor samples inside the same TAD. To be considered altered, a |log2FC|> 1 expression difference was required between the matched grade 2–3 and grade 4 samples. Lowly expressed genes were filtered out so that the remaining genes had over 15 raw counts in at least two grade 4 or grade 2–3 tumors. Genes with rearrangements detected in the same TAD and expression difference in the same direction in at least two patients were reported. Interesting rearrangements were more closely inspected in the aligned WGS BAM files with IGV.

### Public data and analysis

The TCGA and International Cancer Genome Consortium (ICGC) cohorts were analyzed as previously described [23]. The details of their analysis and the analysis of Glioma Longitudinal AnalySiS (GLASS) consortium data from IDHmut astrocytoma cases that progress to grade 4 can be found in the Additional file 2: Supplementary Text.

### Immunohistochemical image analysis

Detailed tumor sample processing and registration of whole-slide images are described in the Additional file 2: Supplementary Text. For the analysis of the multiplex immunohistochemistry (mIHC) images, registered images were processed into hyperstacks, cells were detected with DAPI, and IDH mutation staining was used to detect the staining intensities in tumor cells.

### Survival analysis

Survival analysis was done for the patients diagnosed with a primary low-grade (grade 2–3) IDHmut astrocytoma and with systematically annotated and nonconflicting treatment and survival information available. This resulted in the final cohort of 75 patients, whose primary tumors were treated between 2002 and 2016 at Tays. Oligoastrocytomas lacking 1p19q-codeletion were included in the analysis. If 1p19q status information was not available, all the patients with an oligodendroglioma or oligoastrocytoma diagnosis were excluded. In the relapse-free survival (RFS) analysis, relapse was assumed if the patient was surgically reoperated (n = 38) or died (n = 19). Patients who did not meet the endpoints were censored (n = 18). Only treatment before reoperation or, in the case of no additional operations, death or end of follow-up was considered when grouping patients. For the analysis of progression time, we distinguished progression-free survival, in which progression to grade 4 (based on histopathological analysis upon surgical reoperation, n = 17) and death (n = 25) were considered events, from time to progression, in which only progression to grade 4 was considered an event, and death led to the censoring of the case. Patients who did not meet our endpoints or were missing treatment after relapse or survival information were censored (n = 33 for progression-free survival until progression to grade 4 (P4FS), n = 58 for time to progression to grade 4 (TTP4), and n = 35 for overall survival (OS)). For the P4FS, TTP4, and OS analyses, all treatments until the time of progression were considered when grouping patients. In patient grouping, surgery alone (no postoperative therapy), and surgery with radiation, chemotherapy, or both were distinguished. Survival was visualized with Kaplan–Meier plots, and significance was determined with the log-rank test using the R packages survival (version 3.4–0) and survminer (version 0.4.9). Utilizing the same packages, Cox multivariate proportional hazard models were calculated, using Likelihood Ratio tests. Analysis was performed separately for cases with a primary grade 2 diagnosis, and cases with a primary grade 3 diagnosis.

### Statistical analysis

Statistical testing was performed using R (versions 3.3.5 or 3.4.0). Statistical tests used are described in the text and figure legends. All statistical tests were two-tailed unless otherwise specified.

## Results

**Fig. 1:**
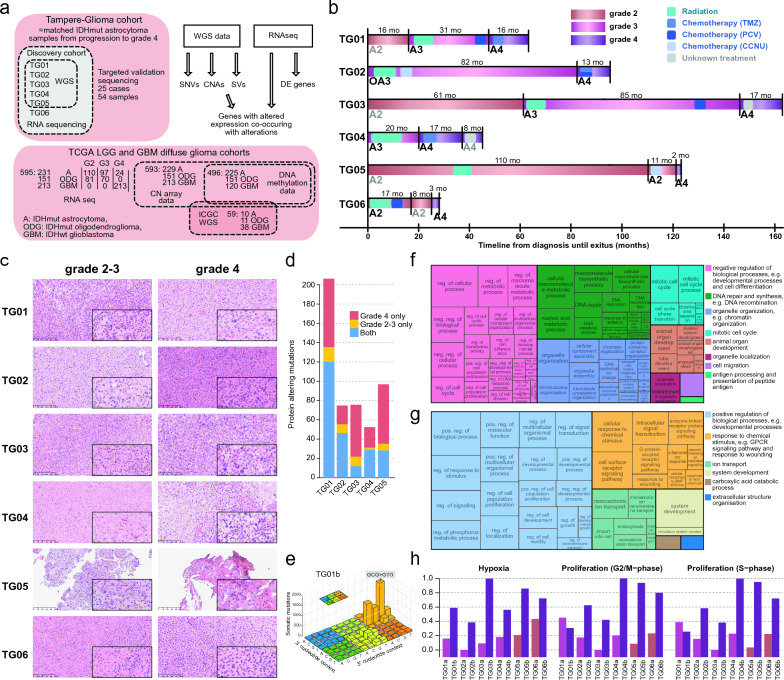
Progression to grade 4 is associated with increased hypoxia response, proliferation, and DNA repair. **a** The discovery cohort comprises six patients with IDHmut low-grade gliomas and relapsed grade 4 tumors. Cases TG01–TG05 were analyzed with WGS and TG01–TG06 with RNA-seq. A larger in-house IDHmut astrocytoma cohort was subjected to targeted DNA-sequencing for validation. In addition, 59 TCGA-ICGC WGS samples, 595 TCGA diffuse glioma RNA-seq samples, and 79 matched GLASS IDHmut astrocytoma samples were used as public validation data cohorts. SNV: single nucleotide variant. **b** Clinical courses of the patients in the discovery cohort. All six patients received radiation treatment and most patients received chemotherapy, but not TMZ, before progressing to grade 4. Vertical black lines represent surgeries and resected samples shown in gray were not measured as part of the discovery cohort. A: astrocytoma, OA: oligoastrocytoma. Tumor type is followed by the tumor grade. **c** Representative H&E images of the tumors in the discovery cohort with scale bars corresponding to 250 µm (low magnification) and 50 µm (high magnification). **d** More protein-altering mutations were detected in TG01 than in other cases (TG02–TG05) in the WGS data. **e** TG01 samples with full *MSH2* deletion and *DNMT3A* inactivation showed a clear CG > TG substitution signature. **f**–**g** GO enrichment analysis for upregulated (**f**) and downregulated (**g**) genes. Upregulated genes were related to DNA repair, cell proliferation, and angiogenesis, whereas downregulated genes were associated with GPCR signaling pathways and the regulation of development. **h** Hypoxia response, cell cycle G2/M-phase, and S-phase gene set Z-score activities were upregulated in grade 4 tumors, except for TG01b, where proliferation activities slightly decreased. Gene set activities were scaled between 0 and 1 for visualization

To better understand the progression of IDHmut astrocytomas to grade 4, we generated and integrated matched WGS and strand-specific RNA-seq data from five Tampere Glioma cohort (TG) patients (TG01–TG05) as well as RNA-seq data from TG06 (Fig. 1a). The key findings were validated using a larger in-house targeted sequencing cohort of 25 patients and published data from TCGA, the ICGC, and the GLASS consortia.

### Clinical course and pathological evaluation of the cases in the discovery cohort

All discovery cohort patients received a primary tumor diagnosis at the age of 25–35 years, except TG01 (age group 18–20) (Fig. 1b, Additional file 2: Fig. S1a and Supplementary Text, Additional file 1: Table S1). Most primary tumors were grade 2 IDHmut astrocytomas; TG02 was initially diagnosed as a grade 3 oligoastrocytoma and TG04 as a grade 3 astrocytoma (Fig. 1b–c, Additional file 2: Fig. S1a–b, Additional file 1: Table S1). All tumors eventually progressed to grade 4 and were at the time diagnosed as secondary glioblastomas. Their diagnosis was later updated to grade 4 astrocytoma based on the WHO 2021 guidelines [3]. Importantly, all patients received radiation during the course of the disease. TG05 was irradiated seven years before the first relapse (due to tumor regrowth based on magnetic resonance imaging (MRI) of the brain), after which chemotherapy alone was administered (Fig. 1b, Additional file 2: Fig. S1a). TG04 was the only patient who received radiation alone before disease progression. For TG01 and TG03, radiation was an initial postoperative treatment (therapy administered between surgeries), followed by chemotherapy closer to the progression than previous surgery. After the initial diagnosis, consecutive cycles of radiation and chemotherapy were administered to patients TG02 and TG06. Before progression to grade 4, patients TG01, TG03, and TG06 received PCV (procarbazine, lomustine, and vincristine) chemotherapy, whereas patients TG02 and TG05 received CCNU (lomustine) chemotherapy. Notably, none of the patients received TMZ before disease progression as it was not part of the standard of care at the time of operation. In surgeries for sequenced grade 2–3 tumors, a residual tumor mass was detected in TG04a and TG05a (Additional file 1: Table S1, Additional file 2: Supplementary Text).

For each of these cases, we analyzed matched tumors before and after progression to grade 4 (Fig. 1b and Additional file 2: Fig. S1a). All tumors harbored a missense mutation in *IDH1* at p.R132 (p.R132G in TG01 and p.R132H in TG02–TG06) (Additional file 1: Table S1), and sample TG05b additionally had a copy-neutral loss-of-heterozygosity in *IDH1* p.R132H. Inactivating *TP53* and *ATRX* alterations were detected in most cases. Progressed grade 4 tumors inherited these driver mutations, consistent with previous reports [24]. No 1p19q co-deletions or *TERT* promoter, *CIC* gene, or *FUBP1* mutations were detected.

Based on DNA methylation analysis, the grade 4 samples belonged to the ‘high-grade astrocytoma’ (A IDH, HG) methylation class (Additional file 2: Fig. S1c and Additional file 1: Table S1) [12]. Many tumors in this class progressed from low-grade astrocytomas. TG01b, TG02b, TG03b, and TG04b had high-confidence classification scores (> 0.84) [25]. TG05b had a score below 0.5 and should generally be discarded if high tumor cell content is assumed [25]; however, it clustered nicely with other A IDH, HG samples in the tSNE plot. To conclude, the samples in our cohort clearly represent IDHmut diffuse astrocytomas.

### *MSH2* and *DNMT3A* inactivation in hypermutator case TG01

To estimate the mutation load in the samples, we counted the protein-altering mutations (excluding known germline variants) in the WGS data. Mutation counts increased, but only moderately, in grade 4 tumors. The total number of coding mutations ranged between 22 and 85 in all tumors except the hypermutator TG01 tumor, which harbored 134 nonsynonymous mutations before progression and 189 mutations in the grade 4 tumor (Fig. 1d and Additional file 1: Table S3). Interestingly, the TG01 sample exhibited a distinct mutational pattern characterized by C > T substitutions (Fig. 1e and Additional file 2: Fig. S1d–e), representing a mixture of SBS1 and SBS15 mutational signatures. SBS1 is observed in many human cancers and is positively correlated with patient age, whereas SBS15 is associated with defective DNA mismatch repair (MMR) and microsatellite instability [20, 26, 27]. The tumors of TG01 harbored inactivating alterations in *MSH2* and *DNMT3A*. *MSH2*, which encodes a member of the DNA MMR complex, was homozygously deleted and markedly downregulated in the TG01 tumor (Additional file 2: Fig. S1f). Furthermore, de novo DNA methyltransferase *DNMT3A* was affected by a p.P904L (chr2:25,234,307:G > A) mutation, disrupting its methyltransferase domain, and loss-of-heterozygosity caused by a large 40 Mb deletion in chromosome 2 (Additional file 1: Table S3). The expression of *DNMT3A* was lower in TG01 than in other cases (Additional file 2: Fig. S1f). DNMT3A p.P904 mutations are rather common and located in the catalytic domain, leading to decreased protein stability [28–30], suggesting defective DNMT3A activity. In both p.P904-mutated TG01 samples, the amino acid substitution was caused by a CG > TG mutation, raising the possibility that these mutations are related to defective DNA MMR.

### Differentially expressed genes are related to proliferation, cell signaling, and development

To examine the changes in tumor cell phenotypes and regulation, we performed pairwise differential gene expression analysis with DESeq2 (adjusted *p* < 0.05, |log2FC| > 1), which resulted in 709 progression-related DE genes (Additional file 1: Table S4). The upregulated genes (n = 386) were linked to the cell cycle, DNA repair, and angiogenesis (ConsensusPathDB and GOrilla gene set enrichment analysis, q < 0.05) (Fig. 1f and Additional file 1: Table S5). Consistently, the progression-related expression signature was dominated by a highly mutually correlated cluster of genes associated with mitotic activity, including the mitotic marker *MKI67* (Additional file 2: Fig. S1g). The downregulated genes (n = 323) were associated with cell differentiation, ion transportation, and G protein-coupled receptor (GPCR) signaling (Fig. 1g and Additional file 1: Table S6). Gene set activity analysis (Additional file 1: Table S2) also supported increased proliferation in all progressed grade 4 tumors, except in TG01b (Fig. 1h). Furthermore, hypoxic response, another marker for higher grade, was elevated in all grade 4 tumors in our data (Fig. 1h).

### Chromosomal rearrangements are increased after tumor progression

**Fig. 2:**
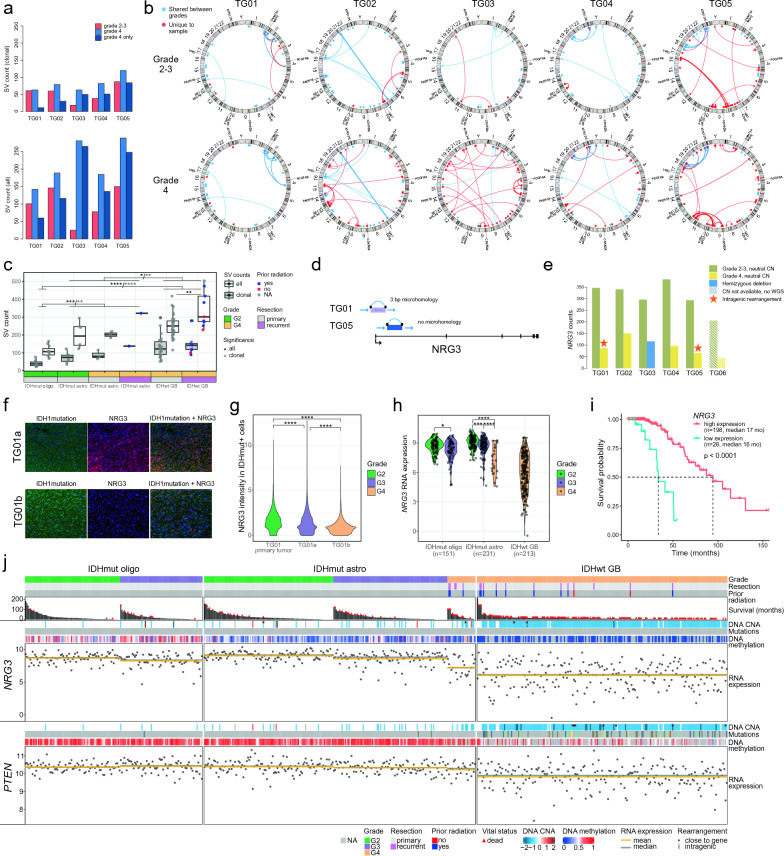
Novel SVs, *NRG3* alterations, and decreased *NRG3* expression were associated with tumor progression. **a** The number of SVs was increased in grade 4 tumors. Clonal SVs (upper panel) were used in the subsequent analyses since they are more likely to contribute to progression. **b** Visualization of clonal SVs with a Circos plot. **c** In the ICGC cohort, the number of SVs was associated with tumor aggressiveness and recurrence. *p < 0.05, **p < 0.01, ***p < 0.001, and ****p < 0.0001 based on Wilcoxon rank-sum test. **d** Intragenic rearrangements hit *NRG3* gene in two grade 4 tumors. The first exon containing the transcription start site was inverted in TG01b, and a section of the first intron was deleted in TG05b. **e** *NRG3* RNA expression decreased in all the tumors upon progression to grade 4 (*p* = 0.0022, Wilcoxon rank-sum test). **f** Representative areas show decreased NRG3 protein expression (red) in IDHmut cells (green) in TG01b. Nuclei are stained with DAPI (blue). **g** NRG3 level in IDHmut cells was decreased upon progression to grade 4. Quantification of mIHC data across cells in three representative areas per sample. The mean expression for the samples was 1.67, 1.40, and 0.94 for the primary TG01, TG01a, and TG01b tumors, respectively. ****p < 0.0001 based on the Wilcoxon rank-sum test. **h** *NRG3* expression was decreased by tumor grade, especially in grade 4 (*p* < 0.0001), in 595 TCGA diffuse glioma cases. *p < 0.05, ***p < 0.001, and ****p < 0.0001 based on the Wilcoxon rank-sum test. **i** Lower *NRG3* RNA expression (below 7.7, mean in the entire TCGA diffuse glioma cohort) was associated with shorter OS in TCGA IDHmut astrocytomas (*p* < 0.0001, log-rank test). **j** In TCGA IDHmut astrocytoma data, *NRG3* was recurrently hemizygously deleted, and its expression was more strongly decreased than *PTEN* in grade 4 IDHmut astrocytomas*.* Rearrangements close to genes (*) were observed in cases with gene loss

For chromosomal rearrangement analysis, we called the SVs from the WGS using an in-house tool Breakfast. To separate rearrangements that were more likely to be clonal from the clearly subclonal ones, we further filtered down the SVs based on the fraction of reads supporting the rearrangement. In grade 2–3 astrocytomas, an average of 53 clonal SVs per sample (range 18–87) was identified (Fig. 2a–b). The number of clonal rearrangements was higher in all grade 4 samples than before progression. In cases TG03–TG05, most rearrangements were unique to grade 4 samples (62–79% of clonal SVs in grade 4 samples) whereas TG01 and TG02 grade 4 tumors inherited the majority of clonal SVs (83% and 62%, respectively) from the lower-grade tumor. The pattern was similar for unfiltered SVs, although higher fractions of SVs (42–94%) were unique to grade 4 tumors (Fig. 2a and Additional file 2: Fig. S2a). This suggests that in cases TG03–TG05, the seeding clone of the progressed tumor had obtained novel rearrangements, whereas many cells and their alterations in grade 2–3 tumors were depleted due to surgery and other treatments. Interestingly, the lowest grade 4-unique SV counts, both filtered and unfiltered, were detected in the hypermutator TG01 tumor. Overall, approximately half (41–67% in clonal and 34–74% in all) of the rearrangements were focal (< 2 Mb), affecting proximal regions of the genome, but also interchromosomal translocations were detected (18–38% of clonal and 20–43% of all rearrangements) (Fig. 2b and Additional file 2: Fig. S2a).

A similar rearrangement analysis was performed for the SV data from the ICGC cohort including both primary and recurrent diffuse gliomas. Among IDHmut astrocytomas, the highest number of SVs was detected in a recurrent grade 4 tumor with prior radiation, whereas SV counts were similar in primary grade 2 and 4 samples (Fig. 2c). IDHwt GBs had significantly higher clonal SV counts than IDHmut tumors (*p* = 6.3*10^–3^ and *p* = 1.6*10^–6^ in respect to IDHmut astrocytomas and oligodendrogliomas, respectively, Wilcoxon rank-sum test), and the counts of all SVs were higher in recurrent than primary GBs (*p* = 0.010, Wilcoxon rank-sum test) (Fig. 2c).

### *NRG3* rearrangements and decreased expression are associated with tumor progression

Chromosomal rearrangements can alter the gene structure or influence gene regulation e.g., by altering regulatory regions or reorganizing TAD structures [31, 32]. TAD boundaries are insulated, and regulatory regions rarely interact with promoters across TAD boundaries [33, 34]. Next, we examined the effects of recurrent chromosomal rearrangements on gene regulation. Fourteen genes were either downregulated (seven genes) or upregulated (seven genes) upon tumor progression, with an accompanying chromosomal rearrangement within the same TAD in at least two cases (Additional file 1: Table S7). For most of these fourteen genes, similar progression-related expression differences were also detected in cases lacking rearrangements (Additional file 1: Table S7). Neuregulin 3 (*NRG3*) gene, located on chr10q23.1, is of particular interest. Progression-related intragenic *NRG3* rearrangements were detected in two cases: a deletion in the first intron in case TG05 and an inversion disrupting the first exon in case TG01 (Fig. 2d); both included putative regulatory regions. Furthermore, TG03b harbored a hemizygous deletion in *NRG3*. *NRG3* was significantly downregulated in all patients upon progression to grade 4 (log2FC = − 3.5, DESeq2 adj. *p* = 4.2*10^–7^, Wald test). Among the cases with WGS data (TG01–TG05), the expression was lowest in cases with rearrangements (Fig. 2e). Downregulation of NRG3 protein in TG01 was also detected by mIHC (Fig. 2f–g). In the ICGC WGS data, additional rearrangements in *NRG3* (one GB) or in the genomic neighborhood of *NRG3* (one grade 2 and one grade 4 IDHmut astrocytoma, one GB) were detected. All these cases also showed a hemizygous loss of *NRG3* and low gene expression (Additional file 2: Fig. S2b).

In TCGA diffuse glioma data (n = 595, Additional file 1: Table S8), *NRG3* RNA expression decreased with tumor grade in IDHmut astrocytomas and oligodendrogliomas (*p* < 0.05, Wilcoxon rank-sum test), especially upon progression to grade 4 astrocytomas (*p* < 0.0001, Wilcoxon rank-sum test) (Fig. 2h). *NRG3* expression was the lowest in IDHwt GBs (Fig. 2h). Furthermore, low *NRG3* expression (below 7.7 i.e. mean expression in the whole TCGA diffuse glioma cohort) was associated with worse survival in the entire diffuse glioma cohort and within IDHmut astrocytomas (*p* < 0.0001, log-rank test) (Fig. 2i and Additional file 2: Fig. S2c). The *NRG3* expression remained significant (hazard ratio 0.56 for the increase of NRG3 expression by its standard deviation, *p* = 0.024, Wald test) in a multivariate Cox proportional hazard model including also patient age and tumor grade as variables (Additional file 1: Table S9), or when using *NRG3* expression alone (hazard ratio 0.49, *p* = 0.0026, Wald test). In IDHmut astrocytomas, hemizygous *NRG3* losses were significantly more frequent in grade 4 than in grade 2–3 tumors (*p* = 1.7*10^–4^, Fisher’s exact test) and were associated with decreased *NRG3* expression (Additional file 2: Fig. S2d). *PTEN*, which is located on the same chromosomal arm as *NRG3*, was also hemizygously deleted in TG03b and TG05b (Additional file 2: Fig. S2e). In TCGA data, *NRG3* was concomitantly deleted with *PTEN* in 30 out of 34 (88%) IDHmut astrocytomas cases with *PTEN* loss, and in all (n = 197) IDHwt GBs with *PTEN* loss (Additional file 1: Table S10). In the GLASS data from matched IDHmut astrocytoma tumors of patients with tumor progression to grade 4 (n = 33, Additional File 1: Table S11), *NRG3* was hemizygously deleted in 14 cases upon progression to grade 4, 13 of which were concomitant with a *PTEN* loss (Additional file 1: Table S12, Additional file 2: Fig. S2f). *NRG3* showed a more dramatic decrease in expression than *PTEN* upon progression to grade 4 in our cases (*p* = 0.0087, Wilcoxon rank-sum test of log2FCs) (Fig. 2e and Additional file 2: Fig. S2e) and in GLASS data (*p* = 0.024, Wilcoxon rank-sum test of log2FCs) (Additional file 2: Fig. S2g). Similarly, the downregulation of *NRG3* was stronger than for *PTEN* in TCGA IDHmut astrocytomas with increasing grade (Fig. 2h,j and Additional file 2: Fig. S2h) and upon hemizygous gene deletion (Additional file 2: Fig. S2d). Besides *NRG3* and *PTEN*, other genes in chromosome 10q are often hemizygously lost. In our data, altogether 170 genes with TCGA RNA and CNA data were downregulated (log2FC < − 1) in the lost regions of chr10 in TG03b and/or TG05b. In TCGA IDHmut astrocytomas, 9 of these genes, including *NRG3*, had significantly (*p* < 0.05, one-sided Wilcoxon rank-sum test, log2FC < − 1) lower expression in cases with hemizygous loss of gene, compared to neutral copy number (Additional file 1: Table S13). Eight of these genes were also significantly downregulated in grade 4 tumors compared to lower grades (*p* < 0.05, Wilcoxon rank-sum test, log2FC < − 1) (Additional file 1: Table S13). To conclude these results, *NRG3* is one of the few genes that is linked to the progression and loss of chromosome 10q in IDHmut astrocytomas.

### Recurrent copy number losses after tumor progression

**Fig. 3:**
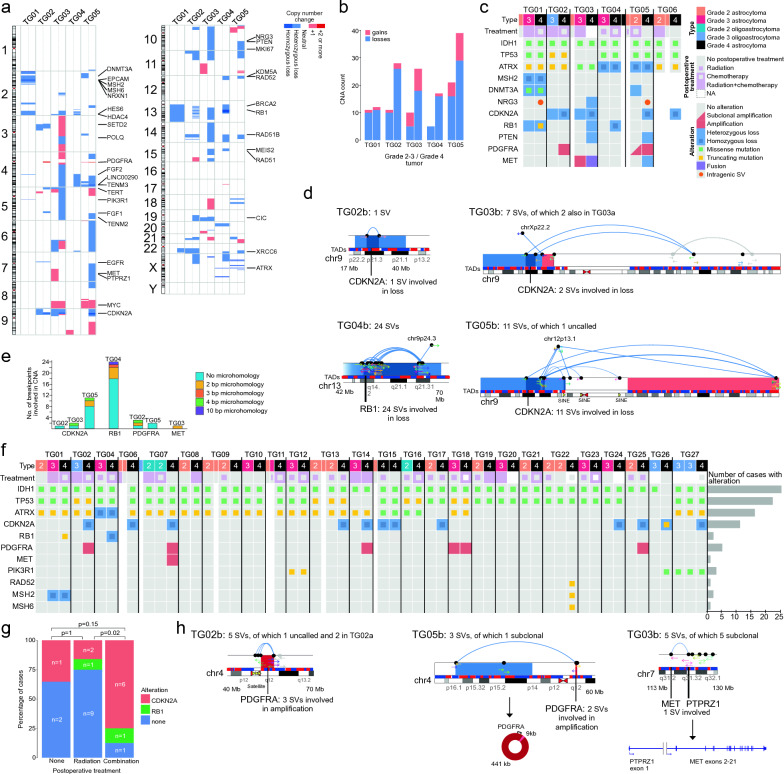
Progression-related *CDKN2A*/*RB1* alterations were associated with postoperative combination therapy and *PDGFRA*/*MET* alterations. **a** DNA copy number patterns of patients TG01–TG05 in grade 2–3 (left column) and grade 4 (right column) tumors. **b** Number of copy number losses was increased upon progression to grade 4, especially in cases without a hypermutator phenotype. **c** Summary of tumor characteristics, patient treatment, and genetic alterations in cases TG01–TG06. Narrow columns represent samples not included in the discovery cohort. **d** *CDKN2A* and *RB1* were homozygously deleted either through simple rearrangements (TG02b) or complex rearrangement patterns. Rearrangements that were already called in grade 2–3 tumors are visualized in gray. Uncalled rearrangements (dashed lines) were aligned to repeat segments (yellow triangles) in TG05b. **e** Short microhomologous sequences were detected in rearrangements involved in the reported DNA copy number alterations. **f** Alterations and treatment information in the targeted sequencing validation cohort consisting of IDHmut astrocytomas, including four discovery cohort cases. Only point mutations, amplifications, and full deletions were analyzed. Narrow columns represent samples with no targeted sequencing data. Relapses after progression to grade 4 are not shown. **g** Patients who underwent both radiation and chemotherapy after surgery were more likely to develop *CDKN2A/RB1* inactivation compared to patients who received radiotherapy alone in the combined discovery and targeted sequencing cohort. Only cases with complete treatment information until progression and confirmed event of inactivation upon progression were included. Inactivating alterations in *CDKN2A* and *RB1* are shown separately but were counted together for Fisher´s exact test. **h** Rearrangement patterns in *PDGFRA* and *MET* genes. The rearrangement at the start of *PDGFRA* amplification in TG02b is located in the centromere and not called because of satellite repeats (yellow triangles). In TG05, *PDGFRA* was amplified as an extrachromosomal DNA. *PTPRZ1-MET* fusion was detected from subclonal rearrangements in TG03b. Uncalled and subclonal rearrangements (dashed arrows and arcs) are only visualized when affecting the alteration

Next, we analyzed DNA copy number losses and gains at a coarse resolution based on WGS coverage. Progression was accompanied by several novel chromosomal gains and losses in all samples except TG01a, in which only one progression-related deletion was detected (Fig. 3a–b). Before progression to grade 4, the highest CNA counts were observed in patient TG05a (Fig. 3b), who had been treated with radiotherapy seven years prior to surgery (Fig. 1b). Notably, losses were more frequent than gains in all grade 2–3 tumors, and their numbers increased more drastically than the gains in progressed grade 4 tumors (Fig. 3b), consistent with previous results that link increased genomic deletions to radiotherapy [8]. A progression-related loss of an overlapping region was detected in at least three cases on chromosomes 4, 9, 13, 14, 22, and X. Only one region harbored recurrent progression-associated gain: the *PDGFRA* locus on chromosome 4.

In ICGC data, IDHwt GBs had a higher proportion of copy number losses than other tumor types (*p* = 0.012 and *p* = 3.4*10^–5^ with respect to IDHmut astrocytomas and oligodendrogliomas, respectively, Wilcoxon rank-sum test). Losses tended to be more frequent than gains in recurrent tumor samples, most of which were taken post-radiation (*p* = 0.14, Wilcoxon rank-sum test) (Additional file 2: Fig. S3a). In contrast, gains were more frequent than losses in primary diffuse glioma tumors (*p* = 0.035, Wilcoxon rank-sum test) (Additional file 2: Fig. S3a).

### Inactivation of *CDKN2A* or *RB1* in progressed grade 4 tumors associated with the combination of chemo- and radiotherapy

Radiation has been reported to increase the risk of *CDKN2A* deletion [8]. Furthermore, homozygous *CDKN2A* deletion has been linked to the recurrence and progression of IDHmut astrocytomas and is currently a determining criterion for a grade 4 diagnosis, irrespective of low-grade tissue histology [3, 10, 35]. Although the original tumor grading was not based on the *CDKN2A* status in our discovery cohort, *CDKN2A* was homozygously deleted and dramatically downregulated in four progressed grade 4 tumors (Fig. 3c and Additional file 2: Fig. S3b). Furthermore, a subclonal truncating mutation (frameshift insertion, chr13:48,307,353:- > AG, p.R71fs, AF 0.20), together with a hemizygous loss of *RB1*, was detected in TG01b and a homozygous deletion of *RB1* in TG04b. In summary, either *CDKN2A* or *RB1* was fully deleted upon progression in all cases, except for the hypermutator TG01 with a subclonal *RB1* inactivation. This makes inactivation of the CDKN2A/RB1 pathway a major mechanism for grade 4 tumor development, at least with the treatment schemes used.

To study the origin of CNAs, we analyzed the rearrangement patterns that caused *CDKN2A* and *RB1* deletions. TG02b had already lost one copy of *CDKN2A* in the first analyzed sample and the second copy was deleted via a simple deletion involving two breakpoints (Fig. 3d). TG03b lost the first copy due to a translocation with chromosome X, and the second *CDKN2A* deletion was related to a fusion between the start of chr9 and an inverted segment (chr9p21.3-chr9q22.33) on the right side of *CDKN2A* (Fig. 3d). In TG05b, the rearrangement pattern was rather complex, involving translocations with chr12 and the end of chr9, thus linking the *CDKN2A* deletion to a large gain in chr9q (Fig. 3d). The rearrangements in TG05b were partly connected to the SINE element AluSx across chromosome 9 and were not reported by the detection tool. Similarly, in TG04b, one copy of *RB1* was lost in a larger deletion, and the second loss was linked to a very complex rearrangement pattern; 24 SVs were detected in the genomic neighborhood and within the depleted region, suggesting that small stretches of DNA were fused with the regions adjacent to the homozygously deleted region. In cases TG03b–TG05b, short (2–4 bp) microhomologous sequences were detected in a fraction of breakpoints (Fig. 3e) and were indicative of MMEJ or NHEJ pathway activity [36]. In one *RB1* rearrangement, the microhomology was 10 bp and was evidently repaired with MMEJ (the sole repair mechanism for breakpoints with 5–18 bp microhomology) [37]. As longer homologies were not detected, HRR was unlikely to be involved.

Targeted DNA sequencing was used to analyze FFPE or fresh frozen tumor material from 21 additional patients with IDHmut astrocytoma who had progressed to grade 4 (Additional file 1: Tables S14-S15). *CDKN2A* was inactivated in the grade 4 tumors of nine (43%) additional patients; it was homozygously deleted in eight cases and carried a truncating mutation together with hemizygous loss in TG26 (Fig. 3f). No additional *RB1* mutations were detected in the targeted sequencing cohort. When considering the inactivation of *CDKN2A/RB1* with respect to treatment, patients who underwent both postoperative radiation and chemotherapy were more likely to develop inactivation after therapy than those who underwent only postoperative radiotherapy (*p* = 0.02, Fisher's exact test) (Fig. 3g). This difference seems unrelated to primary tumor grade (five/six grade 2/3 tumors developed a *CDKN2A/RB1* alteration upon progression to grade 4 vs. six/six grade 2/3 tumors without progression-related alteration, respectively, *p* = 1.0, Fisher’s exact test). Additionally, there were no significant differences in the distribution of grade 2 and 3 tumors regarding the given treatment in this cohort. Only one (TG07) of the seven cases with *CDKN2A/RB1* inactivation after combination therapy received TMZ before the gene inactivation. TP53 is typically defective in IDHmut astrocytomas, which was also true for all but four cases in our discovery and targeted sequencing cohorts (Fig. 3c, f). *TP53* alterations are likely to disturb the TP53-dependent cell cycle checkpoint pathway, induced by DNA damage [38]. In all cases with intact TP53 in our in-house cohorts, progression to grade 4 disease was accompanied by *CDKN2A* inactivation.

As expected, recurrent homozygous deletions in *CDKN2A* and *RB1* were present in TCGA diffuse gliomas and GLASS progressed grade 4 IDHmut astrocytomas (Additional file 1: Table S8 and Table S11 and Additional file 2: Fig. S3c and S2f). In GLASS data, *CDKN2A*/*RB1* inactivation was the most frequent progression-related alteration, with 18 out of 33 analyzed cases having full inactivation of *CDKN2A*, two cases having fully inactivated *RB1*, and one case with a truncating mutation in one *RB1* allele (Additional file 1: Table S12, Additional file 2: Fig. S2f). Subfraction of these gene inactivations were caused by the combined effect of hemizygous loss and a damaging point/frameshift mutation in the other allele. All the damaging point mutations represented the TMZ-associated mutational signature [27] and were detected after TMZ therapy. Both within GLASS and TCGA IDHmut astrocytomas, the inactivation of *CDKN2A* and *RB1* was mainly mutually exclusive. In TCGA, one grade 4 tumor had both *CDKN2A* and *RB1* called as homozygously deleted but only *RB1* showed decreased expression (Additional file 2: Fig. S3c). In GLASS, for one case with a similar alteration pattern in these genes no expression data was available. In TCGA, the majority of grade 4 IDHmut astrocytomas (68%) had a homozygous deletion of *CDKN2A*, which also led to reduced expression at the RNA level. *RB1* was homozygously deleted in three grade 2–3 IDHmut astrocytomas with poor OS rates (Additional file 2: Fig. S3c). *CDKN2A* or *RB1* expression was not associated with survival in diffuse glioma subtypes (Additional file 2: Fig. S3d–e).

### Progression-related activating alterations in growth factor receptor genes concomitant with CDKN2A inactivation

Grade 4 tumors showed mutually exclusive activating alterations in the growth factor receptor genes (Fig. 3c). *PDGFRA* was amplified in TG02b (14 extra copies) and TG05b (50 extra copies). In TG02b, amplification started at a satellite repeat region in the centromere (Fig. 3h). In TG05b, the amplified region included a short 9 kb stretch in chr4p16.1, forming an extrachromosomal DNA amplicon (Fig. 3h). Subclonal *PDGFRA* amplification with identical breakpoints was also detected in TG05a (taken post-radiotherapy), suggesting that this subclone was the seeding clone for the grade 4 tumor. In our targeted sequencing cohort, *PDGFRA* amplification was observed in four additional cases (TG07, TG14, TG18, and TG25) (Fig. 3f). In TG18, amplification was already present in the primary grade 3 tumor and was not coupled with *CDKN2A* deletion; in the others, amplification was acquired upon progression, concomitantly with *CDKN2A* deletion. All these four cases received radiation and two received additional chemotherapy before progression to grade 4. Furthermore, TG03b acquired a subclonal *MET-PTPRZ1* fusion (Fig. 3h). A striking overexpression of the gene was detected in discovery cohort cases with activating alterations in *PDGFRA* or *MET* (Additional file 2: Fig. S3f).

In TCGA IDHmut astrocytomas, *PDGFRA* amplification occurred more often in grade 4 tumors (21% in grade 4, 0.03% in grade 2–3, *p* = 0.0025, Fisher’s exact test) (Additional file 2: Fig. S3c). Even when considering generally higher *PDGFRA* expression in IDHmut astrocytomas than in IDHwt GBs (Additional file 2: Fig. S3c), only a subpopulation of tumors with gene amplification represented outlier expression levels (in IDHmut astrocytomas, four tumors with *PDGFRA* amplification, three of which were grade 4).

Of the 21 additional patients in the targeted sequencing cohort, 11 (52%) showed no *CDKN2A*, *RB1,* or *PDGFRA* alterations. Eight (73%) of these, including TG22 with a truncating mutation in *RAD52*, *MSH2*, and *MSH6* DNA repair genes, received radiation alone before progression to grade 4 (Fig. 3f). Activating *PDGFRA/MET* alterations always co-occurred with a homozygous *CDKN2A* deletion in our discovery cohort and in all but one case in the targeted sequencing cohort (only *PDGFRA* amplification in TG18) (*p* = 0.033 for concomitant *PDGFRA/CDKN2A* alteration in the combined discovery and targeted seq cohort, Fisher’s exact test). Furthermore, *CDKN2A* was completely inactivated in all the grade 4 IDHmut astrocytomas with *PDGFRA* amplification in TCGA and GLASS data with an exception of two cases (one in the GLASS and one in TCGA dataset), which carried only a *PDGFRA* amplification (Additional file 2: Fig. S2f and S3c). However, these two samples also showed no increase in the *PDGFRA* expression (Additional file 2: Fig. S3c, g).

### Progression-related alterations and gene set activities associated with altered DNA repair

**Fig. 4:**
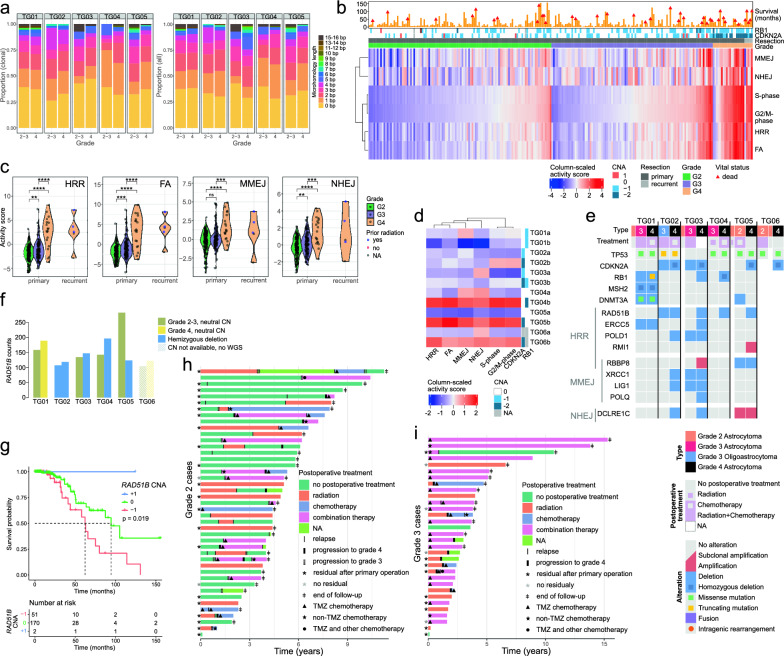
DNA repair is affected upon IDHmut astrocytoma progression. **a** The proportions of different microhomologous sequence lengths in SV breakpoint junctions. Clonal SVs are visualized on the left, and all SVs are on the right. **b** Heatmap of gene set activities in the TCGA IDHmut astrocytomas show distinct activity patterns for MMEJ and NHEJ when compared to proliferation (S- and G2/M-phase). Samples are ordered based on the G2/M gene set activity in each tumor group. **c** DNA repair gene set activities increase with increasing grade in TCGA IDHmut astrocytomas, especially in grade 4. **p < 0.01, ***p < 0.001, and ****p < 0.0001, Wilcoxon rank-sum test. **d** DNA repair and proliferation gene set activities were increased upon progression, especially in TG04–TG06, in our discovery cohort. The DNA copy number status of *CDKN2A* and *RB1* is marked on the right side of the heatmap. **e** Oncoprint showing alterations in DNA repair-related genes with progression-related alterations associated with gene expression in our and the TCGA data. **f** *RAD51B* expression was higher in TG05a, which was collected after radiation, compared to the grade 4 tumors that were also taken post-radiation. *RAD51B* expression was decreased and gene hemizygously deleted in grade 4 TG05b tumor. **g** Hemizygous loss of *RAD51B* was associated with worse OS in the TCGA IDHmut astrocytomas (*p* = 0.019, log-rank test). **h** Heterogeneous treatment paths in grade 2 cohort. The survival cohort comprises 43 patients diagnosed with a grade 2 IDHmut astrocytoma, 29 of which were surgically reoperated and nine progressed to grade 4. Residual tumor information after primary operation (*) was available from 23 patients. **i** Patients with primary grade 3 IDHmut astrocytoma were mainly treated with postoperative radiation or combination therapy. Timelines of 32 patients, 10 of which were treated surgically for relapse and eight progressed to grade 4. Residual tumor information after primary operation (*) was available from 13 patients

Our RNA-seq results suggested that DNA repair pathways are altered in progressed tumors, which have overcome radiotherapy ± chemotherapy-induced selection pressure. DE genes were associated with DNA repair (Fig. 1f), and the number of chromosomal rearrangements and CNAs increased in all grade 4 tumors except for TG01b, which had a defective DNA MMR (Figs. 2a and 3b). Furthermore, only short or no microhomologous sequences were detected at SV breakpoint junctions (Fig. 4a and Additional file 2: Fig. S4a) or were associated with recurrently altered genes (Fig. 3e), suggesting that the SVs were generated by NHEJ or MMEJ activity. None of the microhomologies were longer than 16 bp (Fig. 4a).

We investigated whether the increased number of rearrangements was caused by a defective HRR. *POLQ* and *BRCA2*, which have been used as markers of defective HRR-mediated DNA repair [39, 40], were upregulated (*p* < 0.05, Wilcoxon rank-sum test) upon progression in all discovery cohort cases, except for the hypermutator TG01 case (Additional file 2: Fig. S4b–c). However, their expression was highly correlated with the cell proliferation rate in both our data and the TCGA data (Additional file 2: Fig. S4d), with the exception of *POLQ* in TG03b, which also lost one copy of the gene (Additional file 2: Fig. S4b). Analysis of the overall activity of the genes in DNA repair pathways (Additional file 1: Table S16) showed that gene set activities of HRR and FA pathways, both active especially during mitosis [41], markedly correlated with the cell proliferation rate in the TCGA data, whereas NHEJ and MMEJ gene sets showed more distinct activity profiles (Fig. 4b and Additional file 2: Fig.S4e). For all these gene sets, high activity scores became more prominent with increasing tumor grade (Fig. 4c and Additional file 2: Fig. S4f).

In our discovery cohort, TG01b had the lowest NHEJ and MMEJ gene set activities, which was also evident from the expression levels of several key members of these pathways (Fig. 4d and Additional file 2: Fig. S4b, g–h). In contrast, the activities of these gene sets were clearly upregulated in TG04–TG06 upon progression to grade 4 (Fig. 4d), together with the accumulation of SVs and increased proliferation.

Based on the WGS analysis, we identified 33 DNA repair pathway genes that were genetically altered upon progression to grade 4 (Additional file 2: Fig. S4i and Additional file 1: Table S17). In nine of those, gene expression was associated with CNA (or intragenic rearrangement) in our data and the TCGA diffuse glioma data, and five of them were involved in the HRR pathway (Fig. 4e–f and Additional file 2: Fig. S4b, h, j–k). Four genes in MMEJ or NHEJ pathways were hemizygously deleted in TG02b and/or TG03b, which also showed lower DNA repair pathway gene set activities than other progressed tumors. The gain and higher expression of NHEJ gene *DCLRE1C* (alias *ARTEMIS*, *SCIDA*, *SNM1C*) in TG05a–b, both collected post-radiation, can promote radioresistance [42–44]. In the TCGA data, *DCLRE1C* expression was positively associated with copy number gain and increased after radiation (Additional file 2: Fig. S4k). GLASS data also shows recurrent copy number alterations in these DNA repair genes in the relapsed tumors (Additional file 1: Table S11).

Interestingly, *RAD51B* was hemizygously deleted in all grade 4 samples and the primary tumor TG02a, but not in the hypermutator case TG01 (Fig. 4e). RAD51B functions in HRR of double-stranded DNA breaks generated by DNA-damaging agents and radiation [45], and it is induced by radiation [46]. RAD51B depletion leads to an increased NHEJ/HRR ratio, thus favoring NHEJ over HRR [47], which increases the risk of chromosomal changes. The highest expression of *RAD51B* was observed in TG05a (Fig. 4f), collected after radiation, and was downregulated (log2FC = − 1.18) in TG05b with *RAD51B* loss, resulting in a similar expression level as in cases lacking prior radiation or carrying a hemizygous loss of the gene (Fig. 4f). In the TCGA data, recurrent hemizygous *RAD51B* losses were detected, including in a recurrent IDHmut astrocytoma case with prior radiation, and led to significantly decreased *RAD51B* expression (Additional file 2: Fig. S4k). In GLASS data, progression related hemizygous losses in *RAD51B* were detected in seven cases, five of which were concomitant with *CDKN2A* inactivation. In IDHmut astrocytomas and all diffuse gliomas, *RAD51B* loss was associated with worse OS (*p* < 0.05, log-rank test) (Fig. 4g and Additional file 2: Fig. S4l). In grade 3–4 IDHmut astrocytomas, loss of *RAD51B* was associated with increased proliferation, which was also partly reflected in the HRR and FA activity scores (Additional file 2: Fig. S4m). Interestingly, a trend toward an increased NHEJ activity score upon *RAD51B* loss was detected across all tumor grades (Additional file 2: Fig. S4m).

### Association of combined radiation and chemotherapy with patient prognosis is tumor grade-dependent

To estimate how treatment affects patient outcomes, we obtained and analyzed the timelines and prognosis of 75 grade 2–3 IDHmut astrocytoma patients treated at Tampere University Hospital with respect to the treatment received before relapse or progression to grade 4 (Fig. 4h–i, Additional file 1: Tables S18–S19). Treatment schemes were different between grade 2 (n = 43) and 3 (n = 32) tumors: the majority of patients with a grade 3 tumor received postoperative combination therapy already after primary surgery, whereas, in the grade 2 cohort, postoperative treatments were mainly administered after relapse (Fig. 4h–i). In these prognosis analysis cohorts, 26 of altogether 32 patients (81%) who were treated with postoperative chemo- or combination therapy before progression to grade 4 (or end of follow-up or death), received TMZ. For grade 3 tumors, RFS was significantly longer after combination therapy than radiation (*p* = 0.0034, log-rank test); there were no grade 2 cases with postoperative combination treatment after primary surgery (Additional file 2: Fig. S4n). Partly due to the delayed postoperative therapy in part of the cases, we next stratified the patients based on treatment until progression to grade 4 or death, whichever occurs first. When looking at P4FS, TTP4 (in which death events were censored), or OS, there were no significant differences in prognosis between postoperative therapy options (none, radiotherapy, combination therapy) in cases with primary grade 2 diagnosis (Fig. 4h–i and Additional file 2: Fig. S4o–q), although a trend to improved OS was observed for postoperative combination therapy compared to radiation (*p* = 0.099, log-rank test). Postoperative combination therapy was never administered before relapse for primary grade 2 tumors, whereas 23% of these patients received radiotherapy already after primary tumor surgery (Fig. 4h), which is likely to affect the results. In cases with primary grade 3 diagnosis, combination therapy was consistently associated with significantly better outcomes than no postoperative therapy (including only two cases) and/or postoperative radiation alone (*p* < 0.05, log-rank test) (Fig. 4i and Additional file 2: Fig. S4o–q), consistent with previous reports [48]. In grade 3, all but one of the eight cases that progressed to grade 4 were treated with postoperative radiotherapy alone. Including the patient age at primary diagnosis as a variable into a multivariate Cox proportional hazard model did not change the results (Likelihood Ratio test) (Additional file 1: Table S20).

## Discussion

This study investigated tumor progression in treated IDHmut astrocytomas by integrating WGS and transcriptome sequencing with histological and clinical data. Progression was accompanied by increased numbers of chromosomal rearrangements and deletions, part of which are likely caused by the treatment administered to patients before tumor progression to grade 4 [8]. The inactivation of the CDKN2A/RB1 cell cycle checkpoint pathway was associated with postoperative combination therapy. Recurrent progression-related *PDGFRA* amplifications were concomitant with acquired *CDKN2A* inactivation. After progression to grade 4, we detected recurrent expression-associated one-copy alterations in *NRG3* and *RAD51B* as well as other genes involved in chromosomal DNA repair.

Our discovery cohort (including TG01-TG06) was moderate in size, largely because of challenges in accessing longitudinal frozen samples from these tumors, which also hampered statistical analyses of our results within this cohort. However, we have complemented it with a larger in-house targeted sequencing cohort and public data from GLASS and TCGA consortia, which allowed us to use statistics, validate our findings, estimate generalizability, and compare our results to those of primary tumors. In addition, the lack of blood-derived control DNA samples prohibited us from conducting some analyses such as genome-wide mutation loads. However, we planned the used analyses and approaches to obtain confident results, for example, by concentrating on progression-related changes and analyzing only protein-altering mutations, as these are rare in germline DNA owing to strong evolutionary selection pressure.

In our analysis, *NRG3* showed recurrent inactivating alterations upon progression, and the gene was uniformly downregulated in grade 4 tumors. NRG3 is a less-studied member of the epidermal growth factor (EGF) ligand family, which binds to ERBB4 (alias HER4) tyrosine kinase receptor, but it less efficiently induces receptor activation than other NRGs [49, 50]. The highest *NRG3* expression is detected in the brain, especially in astrocytes, oligodendrocyte precursors, and neurons (https://www.proteinatlas.org/) [50, 51]. NRG3 has been reported to promote the survival of oligodendrocyte precursors [52], act as a repellent for migrating interneurons [53], and contribute to the growth of cellular processes in radial glia [54]. Germline variants of NRG3 have been linked to mental disorders, such as schizophrenia [53]. Interestingly, NRG3 has been shown to modulate neuronal signaling and these effects are mediated by ERBB4 in a manner that is not dependent on receptor activation [55, 56]. Further studies are needed to better understand the functional role of NRG3 in diffuse astrocytomas. One possibility is that NRG3 is able to suppress or otherwise modulate NRG1-mediated activation of ERBB4-positive cells. In our TCGA data analysis, *NRG3* showed reduced expression in grade 4 tumors, and low expression was associated with worse OS in IDHmut astrocytomas both in univariate and multivariate analysis, consistent with previous reports on its prognostic role in diffuse glioma in general [57–59]. Our results clearly linked the gene to IDHmut astrocytoma progression to grade 4.

Patient TG01 showed homozygous deletion of *MSH2*, inactivation of *DNMT3A*, and high mutation counts representing a mixture of the mutational signatures SBS1 and SBS15. The patient’s young age suggests an age-independent cause of the SBS1-type hypermutation. The detected mutational signature differs from that induced by *MSH2* knockout alone in a cell experiment [60]. However, it is consistent with the most prominent pattern detected in MSH2/MSH6-deficient tumors, which show a higher proportion of SBS1 signatures than MLH1/PMS2-mutant MMR-deficient tumors [61]. Fang et al*.* [61] also reported that the typical mutational pattern of MSH2/MSH6-deficient tumors is due to the defective repair of spontaneous deamination. DNMT3A interacts with the proteins of the deamination repair pathway [62], and its deficiency has been linked to elevated protein-altering and C > T mutation counts in a recurrent GB (operated after radiation and TMZ treatment), as well as defective repair of deaminated CpGs in an IDHmut background [63, 64]. Therefore, *DNMT3A* alterations are likely to contribute to the observed hypermutator phenotype in TG01.

Our results suggest that chromosomal rearrangements-related DNA repair pathways are altered in tumors that have progressed to grade 4 after radiation or both radiation and chemotherapy. The alterations in *RAD51B* and other DNA repair genes were one-copy losses or gains, which is coherent with the important role of DNA repair upon DNA damage or during DNA replication. The results suggest that the alterations mainly shift the balance for the choice of the pathway and this way promote more error-prone repair in TG02-TG05. When looking at the SVs in *CDKN2A*, *RB1*, *PDGFRA,* and *MET* and in general, both NHEJ and MMEJ repair could contribute to their formation. NHEJ and MMEJ are commonly induced in cancer, and both promote the accumulation of chromosomal translocations [37, 65, 66], which also generate our detected progression-related alterations and hence, the progression itself.

Previous reports have shown that IDH1 R132H mutation increases DNA repair in an NRAS G12V-shTP53-shATRX background [67]. However, *IDH1* mutation was also associated with increased expression of cell proliferation-related genes, including genes in the HRR and FA pathways, and a higher proportion of Ki67 + proliferating cells, making it challenging to distinguish between mitosis- and DNA repair-related effects accurately. HRR is active during the S–G2 phase of mitosis [41], but radiation-induced DNA damage is repaired very rapidly in IDHmut cells (gammaH2X foci were cleared in less than 4 h in human/mouse IDHmut cells but took up to 48 h in IDHwt mice) [67], which suggests that other pathways than HRR might be involved. This is further supported by previously reported IDH-mutation-induced HRR defects and increased numbers of DNA double-strand breaks [68]. As low-grade astrocytomas proliferate slower than GBs, for example, they are more likely to receive treatment-induced damage outside mitosis. This can also potentially favor more error-prone DNA repair mechanisms, checkpoint inactivation, and better treatment resistance to radiation.

Case TG01 presented a different route to progression, with only very few CNAs and SVs appearing, despite receiving radiation and, after signs of tumor regrowth in MRI, PCV chemotherapy before progression. Although the exact reasons for the higher chromosomal integrity in TG01 could not be defined within the scope of this study, the results are consistent with the previous notion of the GLASS consortium that the progression of IDHmut astrocytomas follows two distinct routes characterized by either a homozygous deletion of *CDKN2A* or hypermutation which is typically TMZ-induced [10]. The treatment generates selection pressure and eliminates a large number of cells. It is possible that in an *MSH2*/*DNMT3A*-deficient background or during TMZ treatment, cells are more likely to acquire point mutations that generate a selective advantage, whereas in their absence, chromosomal rearrangements and instability facilitate the development of favorable alterations, thus providing a selective advantage. This is also supported by the damaging TMZ-associated point mutations that contribute to progression-related *CDKN2A*/*RB1* inactivation after TMZ therapy in the GLASS data. As radiation and chemotherapy damages DNA, which by definition activates DNA repair pathways, it is also possible that HRR is favored in TG01 cells due to intact *RAD51B* or other mechanism, or cells with defective HRR in an *MSH2*/*DNMT3A*-deficient background undergo negative selection.

Our results suggest that although current therapies induce responses, they also influence tumor evolution. All the patients in our discovery cohort received radiation before disease progression, making radiation an inevitable contributor. Previous reports have supported the double-edged role of radiotherapy, as increased DNA deletion counts, *CDKN2A* deletions [8], and side effects, such as a higher risk of long-term neurological complications [4, 69], have been identified. Furthermore, radiation increases the risk of de novo glioma tumors with *CDKN2A* deletions and *PDGFRA* gains/amplifications [70–74]. Interestingly, we observed that the *CDKN2A* deletions were especially associated with postoperative combination therapy, possibly due to higher selection pressure to inactivate the pathway in this treatment setting than after postoperative radiation alone.

Fully inactivating *RB1* alterations are rarer than *CDKN2A* deletions but affect the same cell cycle checkpoint pathway. *RB1* can also carry point mutations which are often heterozygous and have been suggested to play a less clear role in disease aggressiveness, whereas homozygous *RB1* deletions have been linked to high-grade IDHmut astrocytomas and disease aggressiveness [35]. Based on our results on the expression profile and prognosis of TG04 with full *RB1* deletion, *CDKN2A* and *RB1* full inactivation patterns in TCGA data, and the similar biological roles of CDKN2A and RB1, homozygous deletions or other full inactivating alterations in either are likely to be relevant when considering patient prognosis. Since homozygous *CDKN2A *deletion is already a determining criterion for grade 4 in the WHO 2021 classification [3], the relevance of full *RB1* inactivation as a grading criterion could be also evaluated.

In our real-world survival analysis, patient prognosis was associated with treatment in a tumor grade-specific manner. We acknowledge that the confounding factors can also influence our results. Treatment schemes varied among patients and they have been adjusted over the years, the surgical operation date was used as the date of relapse/progression, and the cohort size was limited. More aggressive treatment is typically used when the predicted prognosis is worse, and postoperative therapy is most likely omitted in case of an assumed better prognosis e.g., due to a small tumor volume and complete resection. However, our results are consistent with previous publications: a meta-analysis of low-grade gliomas (grade 1–2) has reported that radiation improved PFS, but not OS, compared to delayed or no radiation, and similarly, combining chemotherapy with radiation led to improved PFS but not OS compared to radiation alone [75]. On the other hand, in a high-risk IDHmut astrocytoma cohort, PCV chemotherapy administered directly after radiation improved both RFS and OS [76, 77]. It has also been reported that early postoperative radio-chemotherapy improved OS in grade 3 but not in grade 2 IDHmut gliomas [78]. In our study, combination therapy was associated with better survival compared to radiation especially in cases with primary grade 3 diagnosis. Considering that most of these patients received TMZ as chemotherapy, our results are consistent with the results of the CATNON study [48].

Based on the previous publications and our results, it is challenging to precisely evaluate the survival benefit especially for patients with grade 2 IDHmut astrocytoma, which is characterized by a lower proliferation rate than grade 3. However, we cannot rule out the possibility that the moderate survival benefit of combination therapy in grade 2 might be affected by the delayed timing or other confounding factors. For our analyzed cases, radiotherapy was typically administered either directly after the operation or as a response to tumor regrowth based on MRI findings. As astrocytomas were considered less treatment-sensitive and chemotherapy, like PCV, was also associated with toxicity, chemotherapy was often given later during the therapy (e.g. after progression/relapse) or not at all. Accordingly, the first administered treatment for most cases in our discovery cohort (TG01, TG03–TG05) was radiotherapy alone, and chemotherapy was typically administered after further signs of relapse based on imaging or after surgical reoperation. Our results support the current idea that using radiotherapy and chemotherapy close together benefits the patients. Still, the results from ongoing and further randomized trials are needed to define optimal treatment and its timing for grade 2 astrocytoma patients, also when considering alternative treatment options, like targeting IDH mutation with inhibition or vaccination [4].

## Conclusions

In this project, we have comprehensively characterized tumor progression in an IDHmut diffuse astrocytoma discovery cohort and generalized and validated our findings with a targeted sequencing cohort and public data from GLASS, ICGC and TCGA. Genomic data was complemented with rich clinical annotations of patients and their treatment schemes. Our results show that the frequency of inactivating *CDKN2A*/*RB1* alterations is highest after tumor progression following post-operative combination therapy, and *PDGFRA*/*MET* alterations co-appear with *CDKN2A* inactivation. We also linked *NRG3* to tumor progression and tumor aggressiveness in diffuse astrocytomas. In the context of DNA repair, our analysis reveals recurrent alterations in *RAD51B* and other DNA repair pathway genes in relapsed and progressed tumors, association of *RAD15B* loss with poor overall survival, and increased gene set activities of NHEJ and MMEJ pathways in high-grade and progressed tumors. Chromosomal rearrangements were increased after progression. These results suggest higher activity of NHEJ and MMEJ pathways in the progressed tumors, which is able to fasten tumor evolution via new chromosomal alterations and can potentially also facilitate tumor progression. The described process is likely to be further influenced by treatment-induced DNA damage that these pathways are responsible for repairing. In summary, our results can lead to a better understanding of poor outcomes and progression-related alterations in IDHmut diffuse astrocytomas in the context of given therapy that contributes to tumor evolution.

### Supplementary Information

**Additional file 1.** Supplementary Tables

**Additional file 2.** Supplementary Figures and Text

## Data Availability

Normalized gene expression counts and somatic protein-altering mutations are available in GEO with an Accession Number GSE233034. The raw data from datasets generated during the current study are not publicly available due lack of patient consent that separately gives permission to this, but the corresponding author can be contacted upon reasonable request. Normalized Illumina HiSeq gene expression counts and Illumina Human Methylation 450 beta values for the TCGA-GBM and TCGA-LGG projects used in this study were downloaded from the public GDC legacy data portal (https://portal.gdc.cancer.gov/legacy-archive/search/f) using the R library (https://bioconductor.org/packages/release/bioc/html/TCGAbiolinks.html). Copy number alterations and driver mutations in TCGA-GBM and TCGA-LGG cohorts can be accessed through cBioPortal (https://www.cbioportal.org/study/summary?id=lgggbm_tcga_pub). TCGA-GBM and TCGA-LGG structural variants from the DKFZ/EMBL variant calling pipeline can be retrieved from the ICGC data portal (https://dcc.icgc.org/repositories). GLASS data were obtained from the GLASS Data Resource (https://www.synapse.org/#!Synapse:syn17038081/wiki/585622).

## Abbreviations

CCNU: Lomustine
CN: Copy number
CNA: Copy number alteration
DE: Differential expression
FA: Fanconi anemia
FFPE: Formalin-fixed, paraffin-embedded
GB: Glioblastoma
GLASS: Glioma Lognitudinal Analysis
GO: Gene ontology
GPCR: G protein-coupled receptor
H&E: Hematoxylin and eosin
HRR: Homologous recombination repair
ICGC: International Cancer Genome Consortium
IDH: Isocitrate dehydrogenase
IDHmut: Isocitrate dehydrogenase mutant
IDHwt: Isocitrate dehydrogenase wild-type
Indels: Insertions and deletions
log2FC: Log2 fold change
mIHC: Multiplex immunohistochemistry
MMEJ: Microhomology-mediated end-joining
MMR: Mismatch repair
MRI: Magnetic resonance imaging
NHEJ: Non-homologous end-joining
NRG3: Neuregulin 3
OS: Overall survival
p: P-value
P4FS: Progression-free survival until progression to grade 4
PCV: Procarbazine, lomustine and vincristine
q: Adjusted p-value, q-value
RFS: Relapse-free survival
RNA-seq: RNA-sequencing
SNV: Single nucleotide variant
SV: Structural variant
TAD: Topologically associating domain
TCGA: The Cancer Genome Atlas
TG: Tampere glioma cohort
TMZ: Temozolomide
tSNE: T-distributed stochastic neighbor embedding
TTP4: Time to progression to grade 4
VAF: Variant allele fraction
WGS: Whole genome sequencing
WHO: World Health Organization
