# Supplementary material for: Genomic characterization of IDH-mutant astrocytoma progression to grade 4 in the treatment setting
Source: Acta Neuropathol Commun. 2023 Nov 6;11:176. doi: 10.1186/s40478-023-01669-9 (PMC10629206; doi:10.1186/s40478-023-01669-9)
Supplement: Supplementary file 2 — Additional file 2. Supplementary Figures and Text [file 40478_2023_1669_MOESM2_ESM.pdf]

Supplementary Figures and Text

Acta Neuropathologica Communications

Genomic characterization of IDH-mutant astrocytoma progression to grade 4 in the treatment setting

Kirsi J. Rautajoki, Serafiina Jaatinen, Anja Hartewig, Aliisa M. Tiihonen, Matti Annala, Iida Salonen, Masi Valkonen, Vili Simola, Elisa M. Vuorinen, Anni Kivinen, Minna J. Rauhala, Riikka Nurminen, Kendra K. Maass, Sirpa-Liisa Lahtela, Arja Jukkola, Olli Yli-Harja, Pauli Helén, Kristian W. Pajtler, Pekka Ruusuvuori, Joonas Haapasalo, Wei Zhang, Hannu Haapasalo, Matti Nykter

Corresponding author: Kirsi J. Rautajoki, kirsi.rautajoki@tuni.fi, +358 50 318 5819, Tampere University

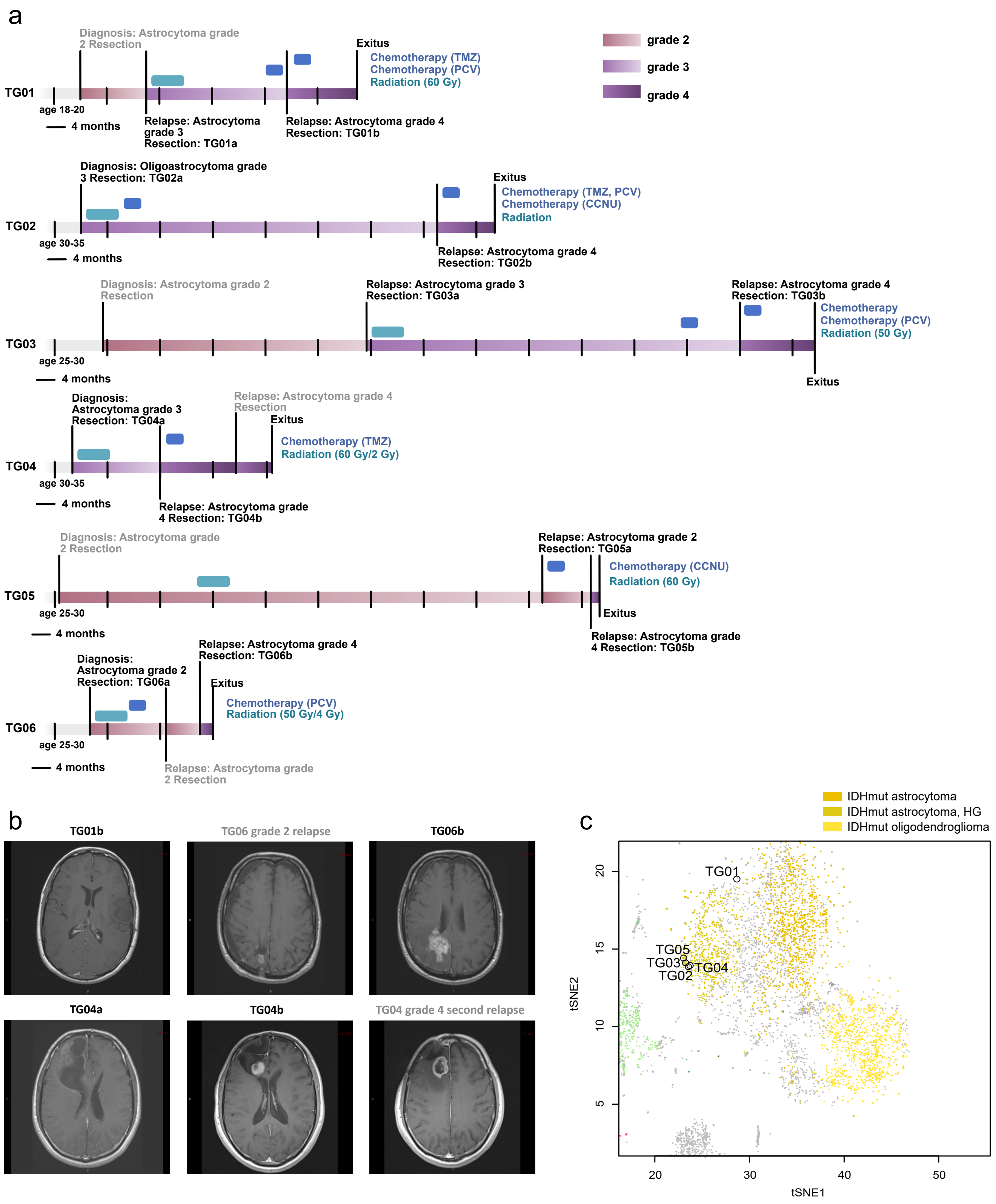

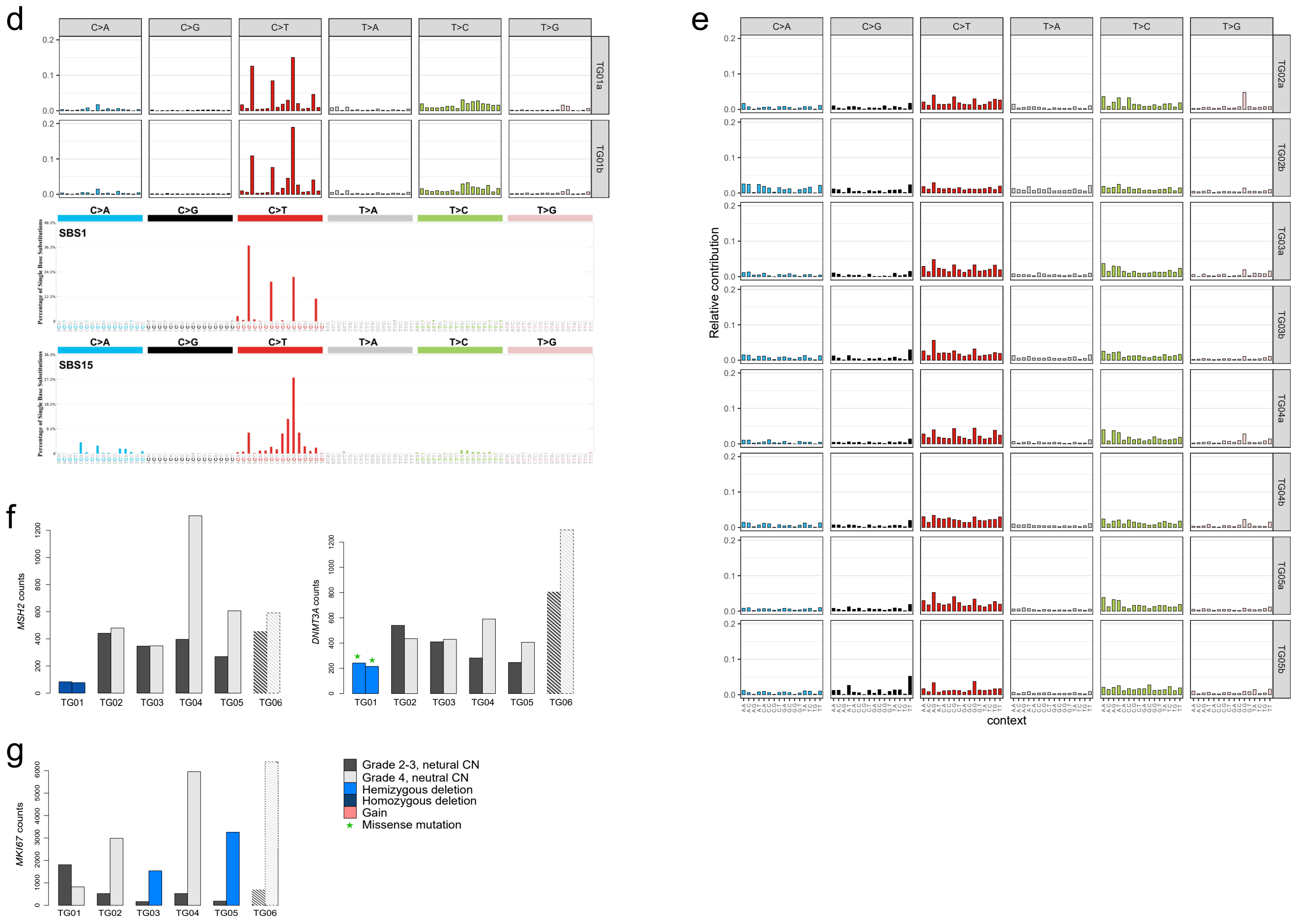

Fig. S1 Additional information on the discovery cohort. **a** Detailed description of the disease course of cases TG01–TG06. **b** MRI scans from selected cases. **c** TG01b–TG05b tumors were positioned next to the high-grade (HG) IDHmut astrocytomas in a t-SNE visualization based on the DNA methylation data. **d** Trinucleotide contexts in TG01a and TG01b show CG>TG signature corresponding to SBS1 and SBS15 (signature figures from: https://cancer.sanger.ac.uk/signatures/sbs/). **e** Cases TG02–TG06 did not show any distinct signature. **f** TG01 showed the lowest expression of *MSH2* and *DNMT3A* of all cases. In TG04–TG06, genes were upregulated upon progression, whereas the expression stayed similar in TG02 and TG03. **g** *MKI67* RNA expression was significantly increased in the grade 4 tumors of patients TG02–TG06 but not in the hypermutator TG01. *MKI67* was hemizygously deleted in cases TG03b and TG05b. CNAs in TG06 (striped bars) are not confirmed due to lack of WGS data.

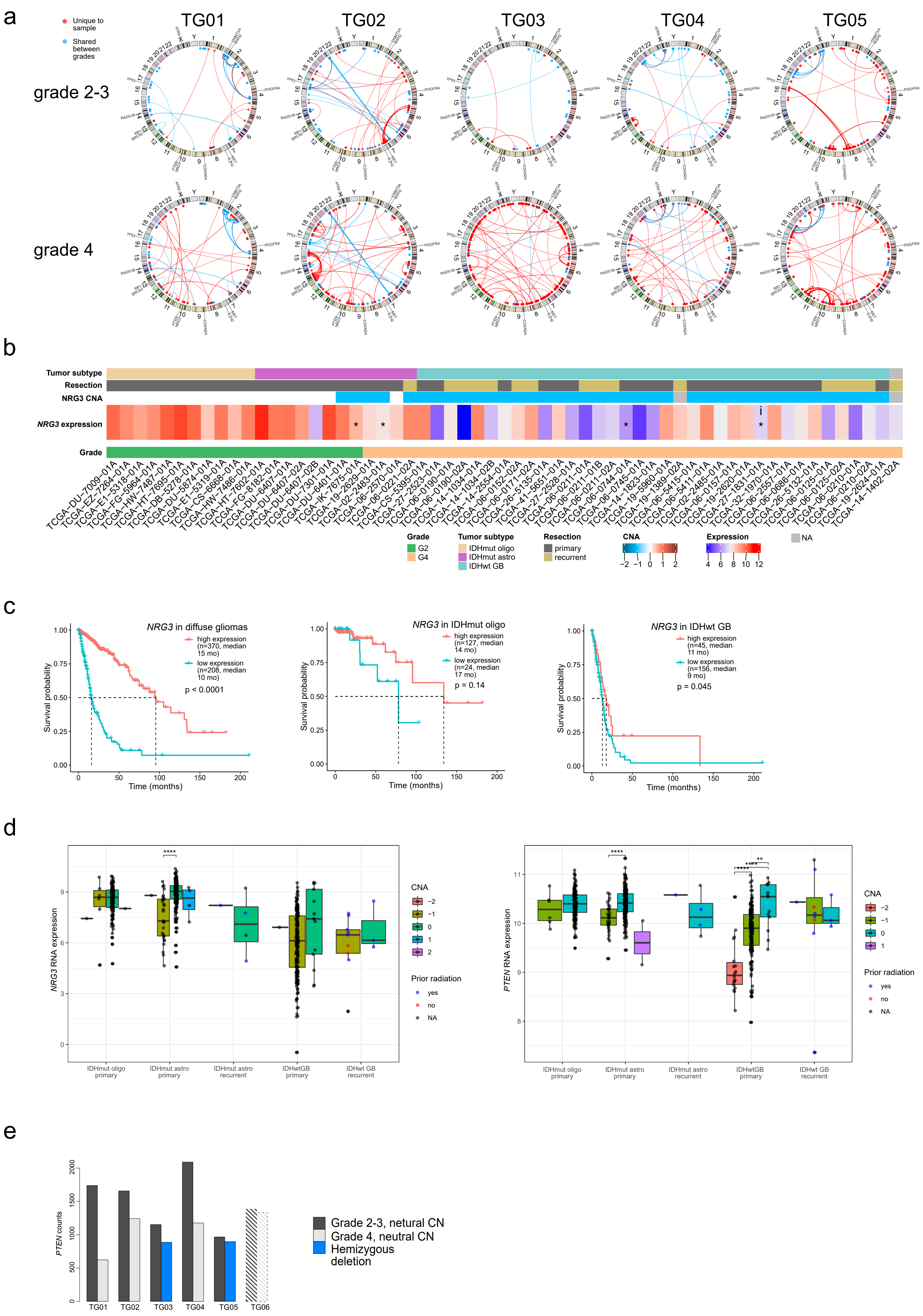

a
Unique to sample
Shared between grades
TG01
TG02
TG03
TG04
TG05
grade 2-3
grade 4
b
Tumor subtype
Resection
NRG3 CNA
NRG3 expression
Grade
Grade
G2
G4
Tumor subtype
IDHmut oligo
IDHmut astro
IDHwt GB
Resection
primary
recurrent
CNA
−2 −1 0 1 2
Expression
4 6 8 10 12
NA
c
NRG3 in diffuse gliomas
high expression (n=370, median 15 mo)
low expression (n=208, median 10 mo)
p < 0.0001
NRG3 in IDHmut oligo
high expression (n=127, median 14 mo)
low expression (n=24, median 17 mo)
p = 0.14
NRG3 in IDHwt GB
high expression (n=45, median 11 mo)
low expression (n=156, median 9 mo)
p = 0.045
Survival probability
Time (months)
d
NRG3 RNA expression
IDHmut oligo primary
IDHmut astro primary
IDHmut astro recurrent
IDHwtGB primary
IDHwt GB recurrent
CNA
Prior radiation
yes
no
NA
PTEN RNA expression
e
PTEN counts
TG01
TG02
TG03
TG04
TG05
TG06
Grade 2-3, netural CN
Grade 4, neutral CN
Hemizygous deletion

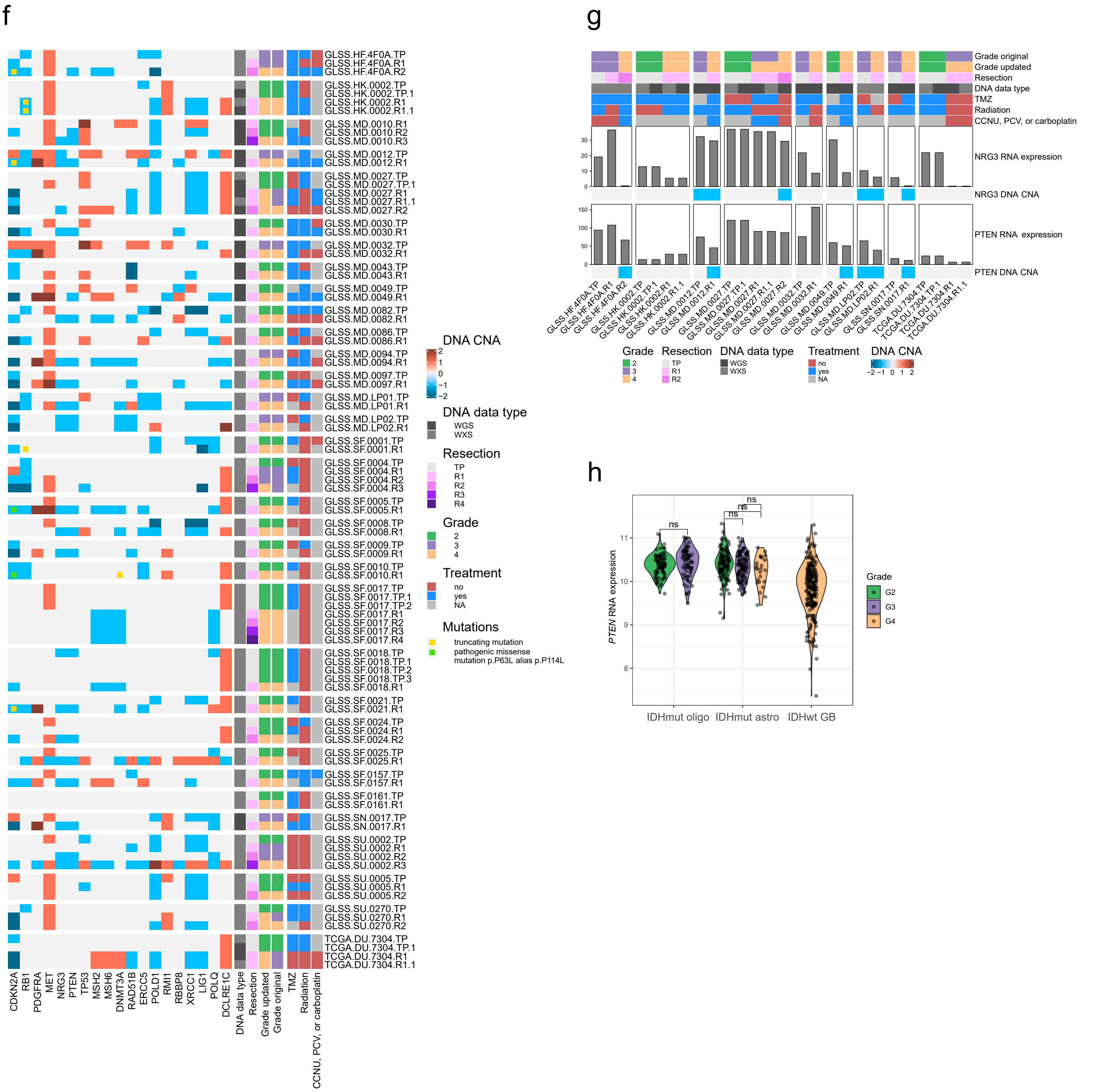

Fig. S2 *NRG3* is recurrently affected by CNAs and SVs in the public validation cohort. **a** Circos plots visualizing all the SVs show an increased number of unique SVs in grade 4 tumors. **b** ICGC copy number calls show recurrent *NRG3* losses, especially in IDHwt GBs. Rearrangements close to *NRG3* (*) were detected in four primary tumors. An intragenic rearrangement (i) was called in a primary IDHwt GB tumor. *NRG3* expression was rather low in cases with rearrangement compared to other cases in the same tumor type. **c** Lower *NRG3* expression (below 7.7, mean in entire TCGA diffuse glioma cohort) was associated with worse survival in diffuse gliomas and in IDHwt tumors (log-rank test). A similar trend was detected in IDHmut oligodendrogliomas. **d** Hemizygous losses of *NRG3* and *PTEN* are associated with decreased expression in primary IDHmut astrocytomas (*NRG3* p=1.6*10-8, *PTEN* p=1.2*10-6, Wilcoxon rank-sum test). **e** *PTEN* expression is lower after progression to grade 4 in discovery cohort (p=0.065, Wilcoxon rank-sum test). **f** Recurrent copy number alterations in GLASS cases progressing to grade 4. Only *CDKN2A*, *RB1* and *DNMT3A* mutations were studied. **g** *NRG3* expression decreases more dramatically in progressed GLASS cases than *PTEN* expression. Samples with CNA data from both WGS and WXS are shown in separate columns. **h** *PTEN* expression is lower in IDHwt GBs than other tumor types in the TCGA data

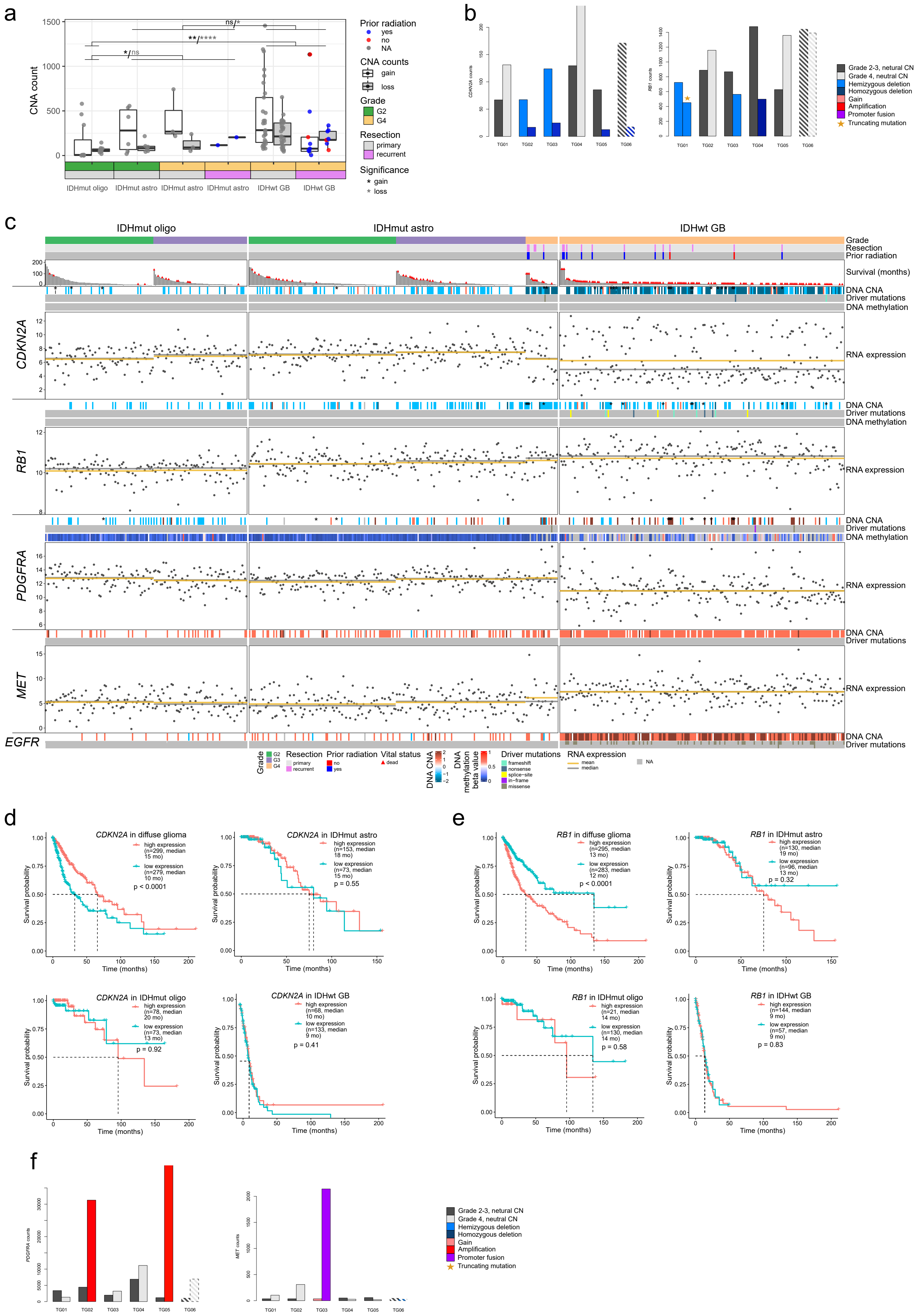
a
CNA count
1500
1000
500
0
ns/*
/**
*/ns
Prior radiation
yes
no
NA
CNA counts
gain
loss
Grade
G2
G4
Resection
primary
recurrent
Significance
* gain
* loss
IDHmut oligo
IDHmut astro
IDHmut astro
IDHmut astro
IDHwt GB
IDHwt GB
b
CDKN2A counts
RB1 counts
TG01
TG02
TG03
TG04
TG05
TG06
Grade 2-3, netural CN
Grade 4, neutral CN
Hemizygous deletion
Homozygous deletion
Gain
Amplification
Promoter fusion
Truncating mutation
c
IDHmut oligo
IDHmut astro
IDHwt GB
Grade
Resection
Prior radiation
Survival (months)
DNA CNA
Driver mutations
DNA methylation
CDKN2A
RNA expression
RB1
PDGFRA
MET
EGFR
Grade
G2
G3
G4
Resection
primary
recurrent
Prior radiation
no
yes
Vital status
dead
DNA CNA
DNA methylation beta value
Driver mutations
frameshift
nonsense
splice-site
in-frame
missense
RNA expression
mean
median
NA
d
CDKN2A in diffuse glioma
high expression (n=299, median 15 mo)
low expression (n=279, median 10 mo)
p < 0.0001
CDKN2A in IDHmut astro
high expression (n=153, median 18 mo)
low expression (n=73, median 15 mo)
p = 0.55
CDKN2A in IDHmut oligo
high expression (n=78, median 20 mo)
low expression (n=73, median 13 mo)
p = 0.92
CDKN2A in IDHwt GB
high expression (n=68, median 10 mo)
low expression (n=133, median 9 mo)
p = 0.41
Survival probability
Time (months)
e
RB1 in diffuse glioma
high expression (n=295, median 13 mo)
low expression (n=283, median 12 mo)
p < 0.0001
RB1 in IDHmut astro
high expression (n=130, median 19 mo)
low expression (n=96, median 13 mo)
p = 0.32
RB1 in IDHmut oligo
high expression (n=21, median 14 mo)
low expression (n=130, median 14 mo)
p = 0.58
RB1 in IDHwt GB
high expression (n=144, median 9 mo)
low expression (n=57, median 9 mo)
p = 0.83
f
PDGFRA counts
MET counts
TG01
TG02
TG03
TG04
TG05
TG06
Grade 2-3, netural CN
Grade 4, neutral CN
Hemizygous deletion
Homozygous deletion
Gain
Amplification
Promoter fusion
Truncating mutation

g

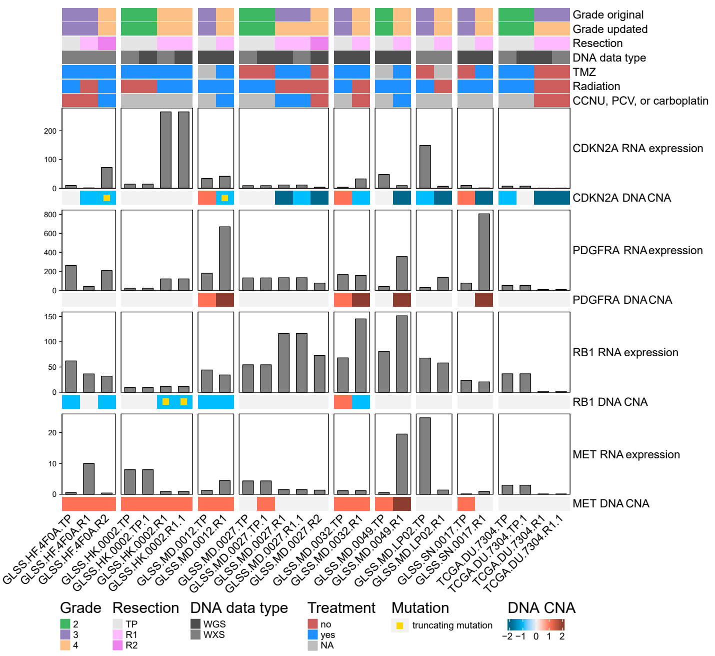

Fig. S3 CNAs in discovery cohort and public validation cohorts. **a** In the ICGC WGS data, CN gains are more common than losses in primary tumors, whereas losses are more frequent in recurrent tumors. IDHwt GBs had more losses than IDHmut tumors and more gains than IDHmut oligodendrogliomas. *p < 0.05, **p < 0.01, and ****p < 0.0001 based on Wilcoxon rank-sum test. **b** Expression of *CDKN2A* and *RB1* follows deletion patterns in our discovery cohort. **c** Summary of alterations in *CDKN2A*, *RB1*, *PDGFRA*, and *MET* in the TCGA diffuse glioma cohort. Asterisks are marking the cases with SVs close to genes, some of which were also intragenic (i). **d** Low *CDKN2A* expression (below mean in entire diffuse glioma cohort) is associated with worse survival in diffuse gliomas in TCGA (log-rank test) but not in the tumor subtypes. **e** High *RB1* expression (below mean in entire diffuse glioma cohort) is significantly associated with worse OS in TCGA entire diffuse glioma cohort (log-rank test) but not in the tumor subtypes. **f** *PDGFRA* is overexpressed in cases with *PDGFRA* amplification. *MET* fusion increases the expression of *MET* in TG03b. **g** In almost all GLASS cases, expression decreases with homozygous deletion in *CDKN2A*. *PDGFRA* and *MET* expression increases with amplification in all but one case. Samples with CNA data from both WGS and WXS are shown in separate columns

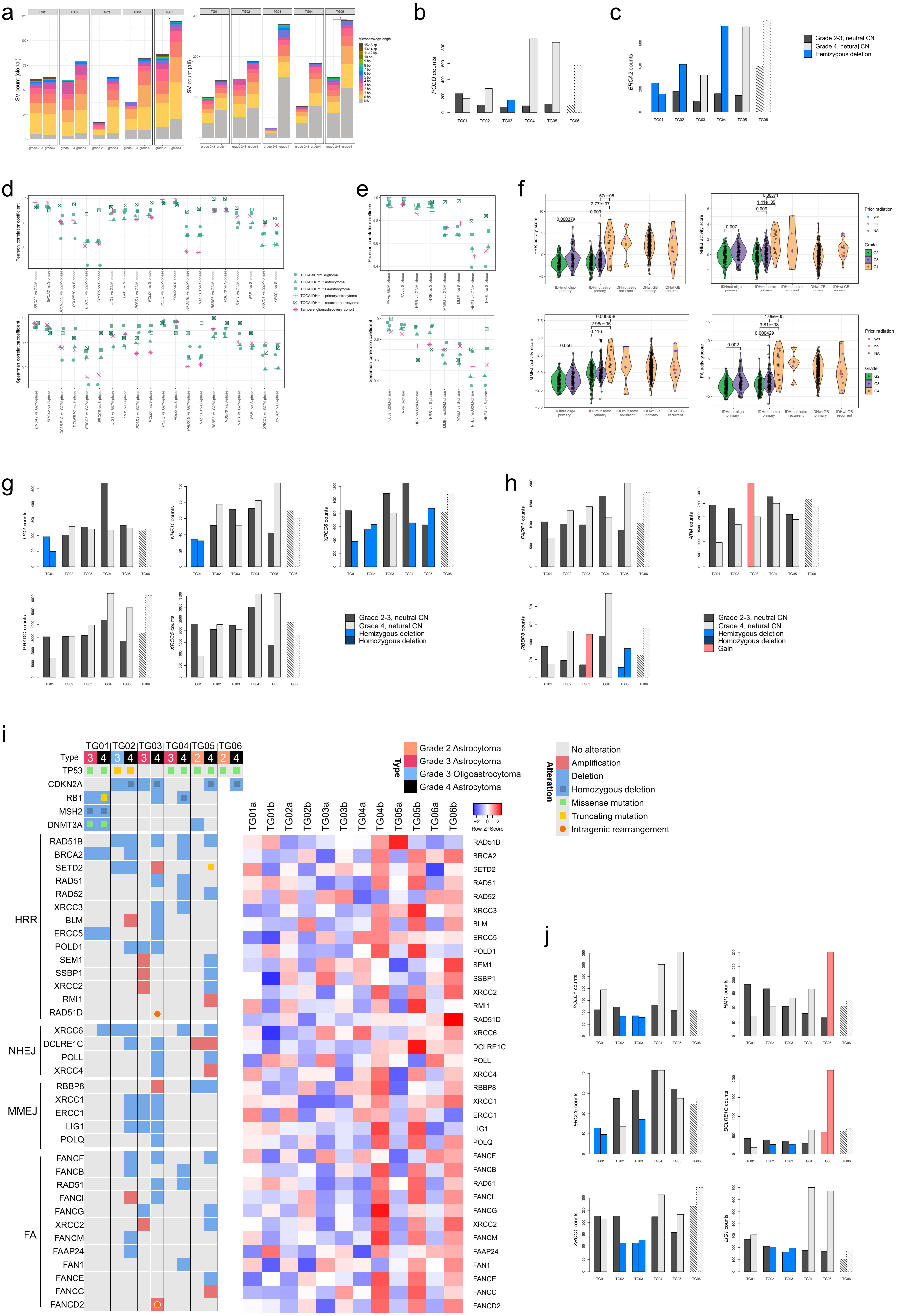
a
SV count (clonal)
SV count (all)
TG01
TG02
TG03
TG04
TG05
grade 2-3
grade4
Microhomology length
15-16 bp
13-14 bp
11-12 bp
10 bp
9 bp
8 bp
7 bp
6 bp
5 bp
4 bp
3 bp
2 bp
1 bp
0 bp
NA
b
POLQ counts
c
BRCA2 counts
Grade 2-3, neutral CN
Grade 4, netural CN
Hemizygous deletion
d
Pearson correlation coefficient
Spearman correlation coefficient
TCGA all diffuseglioma
TCGA IDHmut astrocytoma
TCGA IDHmut G4astrocytoma
TCGA IDHmut primaryastrocytoma
TCGA IDHmut recurrentastrocytoma
Tampere gliomadiscovery cohort
e
f
HRR activity score
NHEJ activity score
MMEJ activity score
FA activity score
IDHmut oligo primary
IDHmut astro primary
IDHmut astro recurrent
IDHwt GB primary
IDHwt GB recurrent
Prior radiation
yes
no
NA
Grade
G2
G3
G4
g
LIG4 counts
NHEJ1 counts
XRCC6 counts
PRKDC counts
XRCC5 counts
Grade 2-3, neutral CN
Grade 4, netural CN
Hemizygous deletion
Homozygous deletion
h
PARP1 counts
ATM counts
RBBP8 counts
Grade 2-3, neutral CN
Grade 4, netural CN
Hemizygous deletion
Homozygous deletion
Gain
i
TG01
TG02
TG03
TG04
TG05
TG06
Type
TP53
CDKN2A
RB1
MSH2
DNMT3A
HRR
RAD51B
BRCA2
SETD2
RAD51
RAD52
XRCC3
BLM
ERCC5
POLD1
SEM1
SSBP1
XRCC2
RMI1
RAD51D
NHEJ
XRCC6
DCLRE1C
POLL
XRCC4
MMEJ
RBBP8
XRCC1
ERCC1
LIG1
POLQ
FA
FANCF
FANCB
RAD51
FANCI
FANCG
XRCC2
FANCM
FAAP24
FAN1
FANCE
FANCC
FANCD2
Type
Grade 2 Astrocytoma
Grade 3 Astrocytoma
Grade 3 Oligoastrocytoma
Grade 4 Astrocytoma
Row Z-Score
Alteration
No alteration
Amplification
Deletion
Homozygous deletion
Missense mutation
Truncating mutation
Intragenic rearrangement
TG01a
TG01b
TG02a
TG02b
TG03a
TG03b
TG04a
TG04b
TG05a
TG05b
TG06a
TG06b
j
POLD1 counts
RMI1 counts
ERCC5 counts
DCLRE1C counts
XRCC1 counts
LIG1 counts

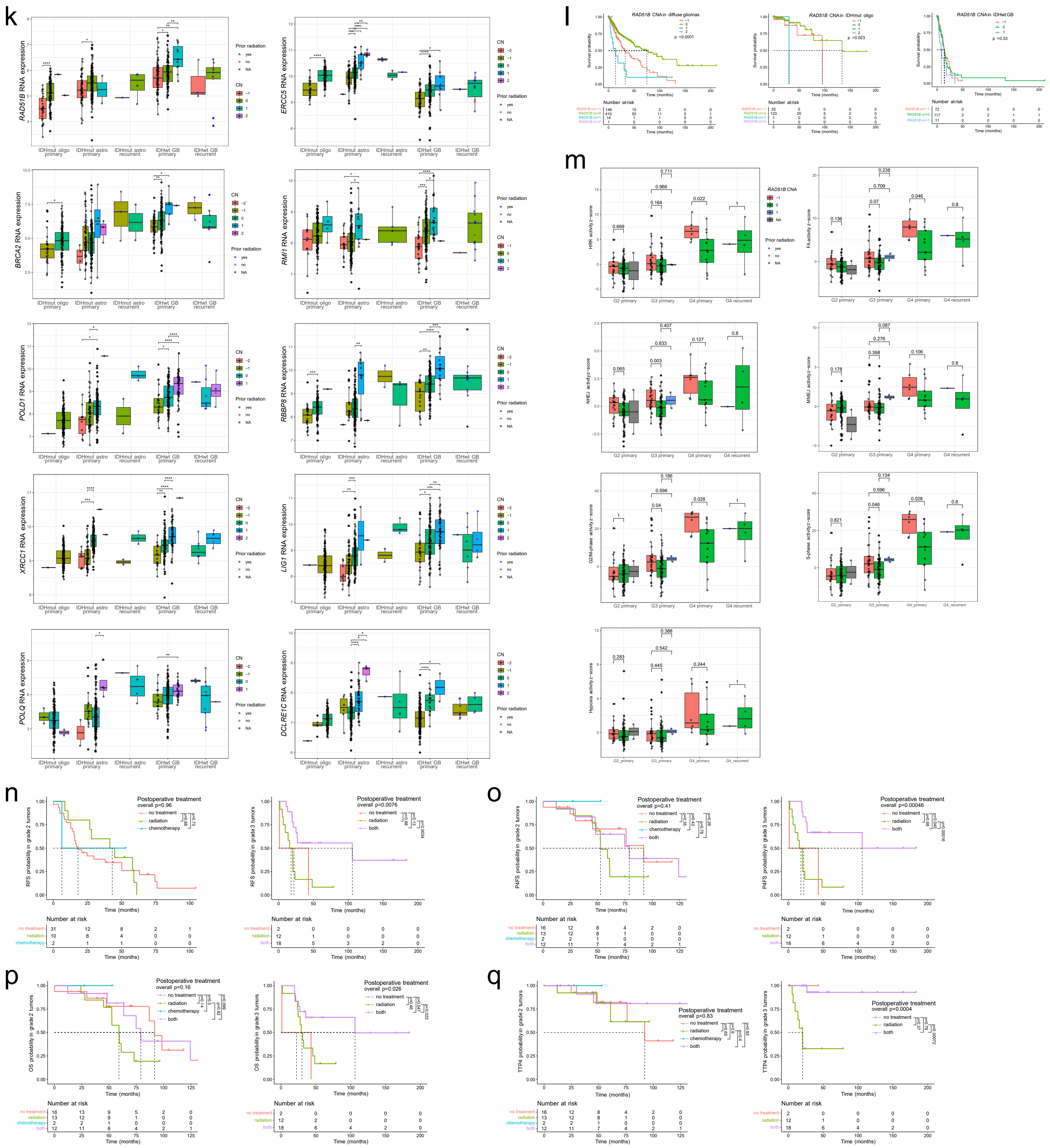

Fig. S4 DNA repair and alterations. **a** Number of SV breakpoint junctions with the microhomologous sequence of different lengths. *p<0.05 based on Fisher's exact test. **b–c** *POLQ* (**b**) and *BRCA2* (**c**) expression in the discovery cohort. **d–e** Correlation of selected genes' expression (**d**) or DNA repair gene sets' activity scores (**e**) with proliferation gene sets' activity scores in the TCGA cohorts and our discovery cohort. NHEJ gene set activity had a higher correlation with cell proliferation in IDHmut grade 4 astrocytomas and recurrent astrocytomas than in other tumor cohorts. **f** DNA repair gene set activity scores in all TCGA diffuse glioma subgroups. p-values based on the Wilcoxon rank-sum test. **g–h** Expression of key NHEJ (**g**) and MMEJ (**h**) genes. **i** Genes in DNA repair pathways with alterations (left) in our discovery cohort and their expression (right). **j** Expression of selected DNA repair genes in our discovery cohort. **k** TCGA expression and CNAs of HRR, MMEJ, and NHEJ genes with progression-related alterations in our discovery cohort. *p<0.05, **p<0.01, ***p<0.001, and ****p<0.0001 based on the Wilcoxon rank-sum test. **l** Hemizygous loss in *RAD51B* was associated with shorter survival in all TCGA diffuse gliomas but not in IDHmut oligodendrogliomas or IDHwt glioblastomas (log-rank test). **m** Gene set activities in TCGA IDHmut astrocytomas stratified by *RAD51B* copy number. p-values based on the Wilcoxon rank-sum test. **n–q** In grade 2 IDHmut astrocytomas postoperative treatment was not associated with RFS (**n**), P4FS (**o**), OS (**p**) or TTP4 (**q**) (log-rank test). In grade 3 tumors, combination therapy is associated with longer RFS (**n**), P4FS (**o**), OS (**p**) and TTP4 (**q**) compared to postoperative radiation therapy (log-rank test)

Supplementary Text

Cases, clinical course, and pathological evaluation

TG01 (age group 18–20 years) was diagnosed with grade 2 astrocytoma (Figure 1b, Figure S1a, and Supplementary Table S1). The patient was reoperated on 15 months later, and the tumor was diagnosed as grade 3 astrocytoma (sample TG01a, Figure 1b–c) with no residual tumor detected. The treatment was continued with radiation (60 Gy). Almost three years later, the patient underwent PCV (procarbazine, lomustine and vincristine) chemotherapy and a third operation for grade 4 astrocytoma (sample TG01b, Figure S1b), followed by temozolomide (TMZ) treatment (five cycles). After surgery, some residual tumor mass was detected by the surgeon. The patient died 16 months after the disease progressed to grade 4 (Figure 1b).

TG02 (age group 30–35 years) was diagnosed with grade 3 oligoastrocytoma after a gross total resection (sample TG02a, Figure 1b–c, and Supplementary Table S1). The surgery was followed by radiation therapy and CCNU (lomustine) chemotherapy. Around seven years later, the patient underwent surgery again, and the tumor was diagnosed as a grade 4 astrocytoma (sample TG02b, Figure 1b–c), followed by TMZ (5 cycles) and PCV chemotherapy. A residual tumor mass was detected after surgery using magnetic resonance imaging (MRI). The patient died 13 months after the grade 4 tumor diagnosis.

TG03 (age group 25–30 years) was diagnosed with grade 2 astrocytoma. No residual tumor was detected after surgical resection. The patient underwent surgery again five years later, and the tumor was diagnosed as a grade 3 astrocytoma after gross total resection (sample TG03a, Figure 1b–c, Supplementary Table S1). Surgery was followed by radiation treatment at 50 Gy. Approximately six years later, tumor growth was detected, and the patient was treated with chemotherapy. Although the response to chemotherapy was good, a residual tumor was detected eight months later. The patient underwent surgery for a third time, and the tumor was diagnosed as grade 4 and could only be partially resected (sample TG03b, Figure 1b–c). Chemotherapy was continued, and the patient died 17 months after the disease progressed.

TG04 (age group 30–35 years) was diagnosed as a grade 3 astrocytoma (sample TG04a, Figure 1b–c, Figure S1b). After surgical resection, a small residual tumor was detected via MRI. Surgery was followed by radiation (60 Gy/2 Gy) (Figure S1a, Supplementary Table S1). Approximately two years later, the patient underwent surgery for the second time, and the tumor progressed to grade 4 (sample TG04b, Figure S1b). A residual tumor mass was suspected by MRI, and treatment was continued with TMZ (eight cycles). One and a half years later, the patient underwent surgery for the third time and received additional treatment (no sequencing or immunohistochemistry (IHC) data were available; Figure S1b). The patient died 25 months after progression to grade 4.

TG05 (age group 25–30 years) was initially diagnosed as a grade 2 ganglioglioma, but the diagnosis was updated to grade 2 astrocytoma after detection of the isocitrate dehydrogenase

(IDH) mutation by IHC staining (no sequencing data available) (Figure S1a, Supplementary Table S1). A residual tumor was detected after surgical resection. Radiation (60 Gy) was administered three years after the primary surgery following an expansion of the residual observed in the computed tomography (CT). Ten years after the original diagnosis, the patient underwent surgery again, and the tumor was diagnosed as grade 2 astrocytoma (sample TG05a, Figure 1b–c). The surgeon detected a small residual tumor mass. The treatment was continued with CCNU chemotherapy. After one year, the patient underwent surgery for the third time, and the tumor was diagnosed as grade 4 (sample TG05b, Figure 1b). Only a partial resection was possible. The tumor tissue was solid and necrotic and contained many blood vessels (Figure 1c). The patient died only two months after progression to grade 4, indicating tumor aggressiveness.

TG06 (age group 25–30 years) was diagnosed as a grade 2 astrocytoma after a gross total resection (sample TG06a, Figure 1b–c). Surgery was followed by radiation treatment (60 Gy/2 Gy) and chemotherapy (six cycles of PCV) (Figure S1a, Supplementary Table S1). One and a half years later, the patient underwent surgery again for grade 2 astrocytoma (Figure S1b). Six months later, the patient underwent surgery for the third time, and the tumor had progressed to grade 4 (sample TG06b, Figure 1b–c, Figure S1b). After surgery, a residual tumor mass was possibly identified by MRI. Two months after surgery, the tumor had spread widely around the brain, and the patient died three months after progression.

Supplementary materials and methods

ICGC structural variant calls

Structural variants called with Delly version 0.6.3 (hg19) were downloaded from the International Cancer Genome Consortium (ICGC) Data Portal for validation analysis, and they included 42 GB and 17 LGG The Cancer Genome Atlas (TCGA) diffuse glioma cohort cases that also were included in the analyzed TCGA RNA-seq cohort. Clonal TCGA rearrangements were filtered by the ratio of rearrangement reads to the total number of reads, which was required to be at least 0.2, either at the junction-spanning reads or within a minimum of four variant read pairs. The same publicly available topologically associating domain (TAD) data were used to determine whether the genes detected in our discovery cohort were rearranged in TCGA.

TCGA RNA-seq, methylation, and survival

Normalized Illumina HiSeq hg19 gene expression counts and Illumina Human Methylation 450 beta values for TCGA-GBM and TCGA-LGG projects were acquired from the National Cancer Institute’s (NCI) Genomic Data Commons (GDC) legacy database through the TCGAbiolinks R library (version 2.18.0) [4, 8, 9]. Genes that had multiple associated methylation probes were filtered based on variance higher than 0.01 between all TCGA methylation samples, including 155 GB and 534 LGG cases. Filtered probes were clustered by Spearman’s correlation distance, and the median beta value of the cluster that correlated negatively with gene

expression in 503 common TCGA samples was chosen to represent gene methylation. According to the WHO 2021 classification of CNS tumors, IDHwt grade 2 and 3 tumors with chromosome 7 gain and chromosome 10 loss, *TERT* promoter mutation, or *EGFR* amplification were considered IDHwt GBs [12], leaving a total of 595 TCGA cases with expression data and 496 cases with both expression and methylation data. Significant differences in log2-transformed RNA expression between glioma subtypes or tumor grades in 595 TCGA cases (151 IDHmut oligodendrogliomas, 231 IDHmut astrocytomas, and 213 IDHwt glioblastomas; Figure 1a) were calculated using the Wilcoxon rank-sum test. The associations between survival and high and low log2 expression status were tested using a log-rank test and visualized with Kaplan-Meier plots using the R packages survival and survminer. Furthermore, a Cox multivariate proportional hazard model with *NRG3* (normalized, untransformed expression divided by its standard deviation used as continuous variable), patient age, and tumor grade as variables was used via R package survival. Patients with recurrent tumors were excluded from the survival analysis.

TCGA CNAs

The copy number alterations (CNAs) and driver mutations for the merged TCGA-LGG and GBM cohorts [2] were downloaded from cBioPortal v.3.6.15 [3, 5]. Significant differences in the number of CNAs in glioma subtypes or tumor grades were calculated using the Fisher's exact test.The differences between CNA status and log2 gene expression were calculated using the Wilcoxon rank-sum test in 593 TCGA cases. Associations between CNA or driver mutation status and survival were estimated using the same process applied to gene expression.

Single nucleotide variant (SNV) and insertion/deletion (indel) analysis in the ICGC cohort

The SNV and indel calls for the ICGC data were obtained from the DKFZ_SNV_Workflow (version 1.0.0) pipeline [7]. The SNVs and indels were filtered to meet the PASS criteria and annotated using ANNOVAR [11].

GLASS CNAs

The clinical cases included in the Glioma Longitudinal AnalySiS (GLASS) cohort were obtained from the GLASS Data Resource [1] available at https://www.synapse.org/#!Synapse:syn17038081/wiki/585622 and all grade 4 tumors were selected. CNA calls were obtained for tumors with IDHmut as well as 1p/19q non-codel status in at least one of the grade 4 tumors. Only cases with available CNA data in at least one low-grade and one grade 4 tumor were considered. The cases were distinguished between primary grade 4 cases and progressed cases, for which at least one low-grade precursor is known, resulting in 33 progressed cases with 79 samples in the analysis cohort. Tumors with homozygous *CDKN2A* deletion were considered grade 4.

GLASS RNA-seq

TPM-normalized counts were obtained from the GLASS Data Resource [10] for the progressed cases with CNA data, resulting in 9 cases with 20 samples. The difference between NRG3 and PTEN log2FCs was calculated between grade 4 and the previous grade 2-3 sample.

GLASS variants

GLASS variant calls were obtained from the GLASS Data Resource for progressed cases with available CNA data. The variants for the genes CDKN2A, RB1 and DNMT3A were obtained, the allele frequency (ad_alt / ad_alt + ad_ref) calculated and mutations with an AF > 0.0 reported.

IHC

The tumor samples were fixed in 4% phosphate-buffered formaldehyde and processed into paraffin blocks. From each sample, 5μm thick sections were cut and stained with an in-house multiplex-IHC (mIHC) protocol based on Multiple Iterative Labeling by Antibody Neodeposition (MILAN) method by Cattoretti *et al.* (2019). Tissue sections were first treated with heat-induced epitope retrieval (HIER) using Tris-HCl buffer (pH 9.0). Antigens were stained using antibodies against IDH1 R132H (H09, Dianova, 1:20) and NRG3 (JE49-84, Invitrogen, 1:200). Antigen detection was carried out using a fluorescently labeled secondary antibody (AlexaFluor 647 anti-rabbit) and DAPI as a counterstain. Slides were mounted using Dako fluorescent mounting media (S302380-2, Dako) and imaged with NanoZoomer S60 (Hamamatsu) whole-slide scanner. The fluorescent mIHC staining was followed by hematoxylin-eosin (H&E) staining on the same tissue section.

Registration of whole slide images

The registration of whole-slide images (WSI) was achieved by taking all the DAPI images from different staining rounds and assigning one of them as fixed. All other DAPI images were aligned pairwise. The registration of an image pair started by finding SIFT features from an image whose long edge was subsampled to 2048 pixels while maintaining the aspect ratio. Matching features between images were found using the Fast Library for Approximate Nearest Neighbors (FLANN) and transformation parameters were estimated using Random Sample Consensus (RANSAC). The affine transform was then fine-tuned by minimizing the Normalized Gradient Fields (NGF) distance between the images, which aimed to match the orientations of the gradients for the two images, making it suitable for multi-stain registration [6]. This registration process was repeated for three different values of epsilon (10, 60, and 110), which controls the noise level of the gradient magnitudes. Below this value, the gradients have an insignificant effect on the overall score. Among the three registrations obtained, the best was selected by visual inspection. If automatic registration did not produce sufficient accuracy, the images were annotated manually for the three landmark points, and an optimal affine transform in the least-squares sense was applied. Insufficient registration accuracy was detected by visual inspection. The transformations acquired for each DAPI image, whether using an automatic or manual approach, were also applied to the marker images to obtain the registered WSI stacks.

Regions of interest (ROI) points were selected from H&E-stained images utilizing the in-house platform Cytomine and confirmed to be representative of the tumor by a neuropathologist. The obtained coordinates were downloaded, and each registered whole-slide image marker was cropped to form a 5000×5000 pixel image with the selected ROI in the middle. For each tumor, three ROIs were obtained, except for TG05a and TG05b, for which two ROIs were obtained.

Analysis of IHC images

The images were analyzed using ImageJ 2.3.0/1.53q/Java 1.8.0_172 and QuPath version 0.3.2. The registered images were processed into hyperstacks using a custom-written ImageJ Macro. The hyperstacks were loaded into QuPath, and cells were detected using the automated cell detection function based on the DAPI channel ("backgroundRadius":15.0, "medianRadius":0.0, "sigma":3.0, "minArea":10.0, "maxArea":800.0, "threshold":3.0, "watershedPostProcess": true, "cellExpansion": 12.0, "includeNuclei":true, "smoothBoundaries":true, "makeMeasurements": true). All cells with a mean IDH1 intensity (in the cells) above the threshold were considered tumor cells. For each identified tumor cell, marker measurements were exported and the mean intensity of NRG3 across all ROIs in the cytoplasm was plotted.

Supplementary References

1. Barthel FP, Johnson KC, Varn FS, Moskalik AD, Tanner G, Kocakavuk E, Anderson KJ, Abiola O, Aldape K, Alfaro KD, Alpar D, Amin SB, Ashley DM, Bandopadhayay P, Barnholtz-Sloan JS, Beroukhim R, Bock C, Brastianos PK, Brat DJ, Brodbelt AR, Bruns AF, Bulsara KR, Chakrabarty A, Chakravarti A, Chuang JH, Claus EB, Cochran EJ, Connelly J, Costello JF, Finocchiaro G, Fletcher MN, French PJ, Gan HK, Gilbert MR, Gould PV, Grimmer MR, Iavarone A, Ismail A, Jenkinson MD, Khasraw M, Kim H, Kouwenhoven MCM, LaViolette PS, Li M, Lichter P, Ligon KL, Lowman AK, Malta TM, Mazor T, McDonald KL, Molinaro AM, Nam D-H, Nayyar N, Ng HK, Ngan CY, Niclou SP, Niers JM, Noushmehr H, Noorbakhsh J, Ormond DR, Park C-K, Poisson LM, Rabadan R, Radlwimmer B, Rao G, Reifenberger G, Sa JK, Schuster M, Shaw BL, Short SC, Smitt PAS, Sloan AE, Smits M, Suzuki H, Tabatabai G, Van Meir EG, Watts C, Weller M, Wesseling P, Westerman BA, Widhalm G, Woehrer A, Yung WKA, Zadeh G, Huse JT, De Groot JF, Stead LF, Verhaak RGW, GLASS Consortium (2019) Longitudinal molecular trajectories of diffuse glioma in adults. Nature 576:112–120

2. Ceccarelli M, Barthel FP, Malta TM, Sabedot TS, Salama SR, Murray BA, Morozova O, Newton Y, Radenbaugh A, Pagnotta SM, Anjum S, Wang J, Manyam G, Zoppoli P, Ling S, Rao AA, Grifford M, Cherniack AD, Zhang H, Poisson L, Carlotti CG Jr, Tirapelli DP da C, Rao A, Mikkelsen T, Lau CC, Yung WKA, Rabadan R, Huse J, Brat DJ, Lehman NL, Barnholtz-Sloan JS, Zheng S, Hess K, Rao G, Meyerson M, Beroukhim R, Cooper L, Akbani R, Wrensch M, Haussler D, Aldape KD, Laird PW, Gutmann DH, TCGA Research Network, Noushmehr H, Iavarone A, Verhaak RGW (2016) Molecular Profiling Reveals Biologically Discrete Subsets and Pathways of Progression in Diffuse Glioma. Cell 164:550–563

3. Cerami E, Gao J, Dogrusoz U, Gross BE, Sumer SO, Aksoy BA, Jacobsen A, Byrne CJ, Heuer ML, Larsson E, Antipin Y, Reva B, Goldberg AP, Sander C, Schultz N (2012) The cBio cancer genomics portal: an open platform for exploring multidimensional cancer genomics data. Cancer Discov 2:401–404

4. Colaprico A, Silva TC, Olsen C, Garofano L, Cava C, Garolini D, Sabedot TS, Malta TM, Pagnotta SM, Castiglioni I, Ceccarelli M, Bontempi G, Noushmehr H (2016) TCGAbiolinks: an R/Bioconductor package for integrative analysis of TCGA data. Nucleic Acids Res 44:e71

5. Gao J, Aksoy BA, Dogrusoz U, Dresdner G, Gross B, Sumer SO, Sun Y, Jacobsen A, Sinha R, Larsson E, Cerami E, Sander C, Schultz N (2013) Integrative analysis of complex cancer

genomics and clinical profiles using the cBioPortal. Sci Signal 6:l1

6. Haber E, Modersitzki J (2006) Intensity gradient based registration and fusion of multi-modal images. Med Image Comput Comput Assist Interv 9:726–733

7. Jones DTW, Hutter B, Jäger N, Korshunov A, Kool M, Warnatz H-J, Zichner T, Lambert SR, Ryzhova M, Quang DAK, Fontebasso AM, Stütz AM, Hutter S, Zuckermann M, Sturm D, Gronych J, Lasitschka B, Schmidt S, Seker-Cin H, Witt H, Sultan M, Ralser M, Northcott PA, Hovestadt V, Bender S, Pfaff E, Stark S, Faury D, Schwartzentruber J, Majewski J, Weber UD, Zapatka M, Raeder B, Schlesner M, Worth CL, Bartholomae CC, von Kalle C, Imbusch CD, Radomski S, Lawerenz C, van Sluis P, Koster J, Volckmann R, Versteeg R, Lehrach H, Monoranu C, Winkler B, Unterberg A, Herold-Mende C, Milde T, Kulozik AE, Ebinger M, Schuhmann MU, Cho Y-J, Pomeroy SL, von Deimling A, Witt O, Taylor MD, Wolf S, Karajannis MA, Eberhart CG, Scheurlen W, Hasselblatt M, Ligon KL, Kieran MW, Korbel JO, Yaspo M-L, Brors B, Felsberg J, Reifenberger G, Collins VP, Jabado N, Eils R, Lichter P, Pfister SM, International Cancer Genome Consortium PedBrain Tumor Project (2013) Recurrent somatic alterations of FGFR1 and NTRK2 in pilocytic astrocytoma. Nat Genet 45:927–932

8. Mounir M, Lucchetta M, Silva TC, Olsen C, Bontempi G, Chen X, Noushmehr H, Colaprico A, Papaleo E (2019) New functionalities in the TCGAbiolinks package for the study and integration of cancer data from GDC and GTEx. PLoS Comput Biol 15:e1006701

9. Silva TC, Colaprico A, Olsen C, D’Angelo F, Bontempi G, Ceccarelli M, Noushmehr H (2016) TCGA Workflow: Analyze cancer genomics and epigenomics data using Bioconductor packages. F1000Res 5:1542

10. Varn FS, Johnson KC, Martinek J, Huse JT, Nasrallah MP, Wesseling P, Cooper LAD, Malta TM, Wade TE, Sabedot TS, Brat D, Gould PV, Wöehrer A, Aldape K, Ismail A, Sivajothi SK, Barthel FP, Kim H, Kocakavuk E, Ahmed N, White K, Datta I, Moon H-E, Pollock S, Goldfarb C, Lee G-H, Garofano L, Anderson KJ, Nehar-Belaid D, Barnholtz-Sloan JS, Bakas S, Byrne AT, D’Angelo F, Gan HK, Khasraw M, Migliozzi S, Ormond DR, Paek SH, Van Meir EG, Walenkamp AME, Watts C, Weiss T, Weller M, Palucka K, Stead LF, Poisson LM, Noushmehr H, Iavarone A, Verhaak RGW, GLASS Consortium (2022) Glioma progression is shaped by genetic evolution and microenvironment interactions. Cell. doi: 10.1016/j.cell.2022.04.038

11. Wang K, Li M, Hakonarson H (2010) ANNOVAR: functional annotation of genetic variants from high-throughput sequencing data. Nucleic Acids Res 38:e164

12. (2021) WHO Classification of Tumours Editorial Board. Central nervous system tumours: WHO classification of tumours series, 5th edition. International Agency for Research on Cancer, Lyon
